# Composite Biomarkers Derived from Micro-Electrode Array Measurements and Computer Simulations Improve the Classification of Drug-Induced Channel Block

**DOI:** 10.3389/fphys.2017.01096

**Published:** 2018-01-04

**Authors:** Eliott Tixier, Fabien Raphel, Damiano Lombardi, Jean-Frédéric Gerbeau

**Affiliations:** ^1^Inria Paris, Paris, France; ^2^Sorbonne Universités, Université Pierre et Marie Curie—Paris 6, UMR 7598 LJLL, Paris, France

**Keywords:** cardiac electrophysiology, numerical simulations, bidomain model, micro-electrode array, classification, drug safety evaluation

## Abstract

The Micro-Electrode Array (MEA) device enables high-throughput electrophysiology measurements that are less labor-intensive than patch-clamp based techniques. Combined with human-induced pluripotent stem cells cardiomyocytes (hiPSC-CM), it represents a new and promising paradigm for automated and accurate *in vitro* drug safety evaluation. In this article, the following question is addressed: which features of the MEA signals should be measured to better classify the effects of drugs? A framework for the classification of drugs using MEA measurements is proposed. The classification is based on the ion channels blockades induced by the drugs. It relies on an *in silico* electrophysiology model of the MEA, a feature selection algorithm and automatic classification tools. An *in silico* model of the MEA is developed and is used to generate synthetic measurements. An algorithm that extracts MEA measurements features designed to perform well in a classification context is described. These features are called composite biomarkers. A state-of-the-art machine learning program is used to carry out the classification of drugs using experimental MEA measurements. The experiments are carried out using five different drugs: mexiletine, flecainide, diltiazem, moxifloxacin, and dofetilide. We show that the composite biomarkers outperform the classical ones in different classification scenarios. We show that using both synthetic and experimental MEA measurements improves the robustness of the composite biomarkers and that the classification scores are increased.

## Introduction

One of the main goals of safety pharmacology studies is to anticipate how drugs affect cardiomyocytes. Among other adverse effects, it focuses on predicting arrhythmic behaviors which may lead to torsades de pointes (TdP). The most common risk factors under consideration are QT prolongation and hERG block. However these risk factors are now considered insufficient and the guidelines need to be improved (Fermini et al., 2016). For instance, an observed QT prolongation is not necessarily associated with TdP risk (Antzelevitch et al., 2004). Several advances in technology and computational modeling may favor the emergence of new methods for more efficient drug safety evaluation. On the hardware side, the Micro-Electrode Array (MEA) technology^1^ (Meyer et al., 2004) enables high-throughput electrophysiology measurements that are less labor-intensive than patch-clamp based techniques. This device has been successfully used in large drug studies (Blinova et al., 2016). On the biological side, the use of human-induced pluripotent stem cells (hiPSC) has developed (Scott et al., 2013) and their recent large-scale production makes it a viable human model replacement. The combined use of the MEA technology and hiPSC cardiomyocytes (hiPSC-CM) represents a new and promising paradigm for automated and accurate *in vitro* drug safety evaluation (Clements and Thomas, 2014; Cavero et al., 2016). The CIPA initiative (Cavero et al., 2016; Fermini et al., 2016) promotes disruptive drug safety guidelines, in particular the use of hiPSC-CM and *in silico* modeling. In parallel of these technological breakthroughs, several efforts have been recently made toward promoting the use of computational tools in drug safety evaluation (Davies et al., 2016; Lancaster and Sobie, 2016). In this context, a framework for drug safety evaluation using *in silico* models and experimental measurements using a MEA device is hereby presented. The device considered in the present work is a six-well nine-electrode MEA but, as shown in Raphel et al. (2017), the approach is general enough to be extended to other types of MEA.

The framework aims at predicting the effect of a drug onto the cardiomyocytes ion channels activities from the knowledge of MEA experimental recordings. More precisely, the goal is to determine which ion channels are affected by a given drug. Note that the aim of the present study is not to predict the drugs propensities to induce cardiac arrhythmias but rather to identify which ion channel is primarily blocked. This represents a first step toward the use of the MEA-hiPSC-CM platform in arrhythmogenicity studies. Even though patch-clamp experiments are the gold standard to assess drug-induced channel block, it was shown in a recent study (Raphel et al., 2017) that it is possible to do so also using MEA field potential measurements. The approach is based on an *in silico* model of the MEA and the hiPSC-CM tissue, a feature selection algorithm and a classification model. The *in silico* model is based on a simple ionic model (Bueno-Orovio et al., 2008) for the cardiomyocytes electrical activity and on the bidomain equations (Tung, 1978) for the spatial propagation of the electrical potentials. The ionic model counts three different currents (fast inward, slow inward, slow outward), each being associated with an ionic species (respectively sodium, calcium, potassium). The activity of each current is controlled by a scaling parameter that is referred to as conductance in the following. In the present work, the drugs considered are assumed to affect one of those three currents. Thus, the inactivation of a current caused by a drug is modeled by a diminution of the corresponding conductance in the ionic model. The conductances and some other parameters of the model can be varied in order to replicate the variability observed in the experimental measurements. The *in silico* model is used to generate what is later referred to as synthetic MEA measurements. The experimental data set itself consists of MEA electrode recordings which come in the form of time series. Each recording is done in control conditions (no drug) and with different drug concentration levels. The experimental data is also labeled, meaning the affected ionic channels are known for each drug.

As explained above, the MEA measurements, whether synthetic or experimental, come in the form of time series. For classification purposes, it is more efficient to extract features from these time series. Some features, also called biomarkers, are already widely used in the community such as the field potential duration (Clements and Thomas, 2014) which may be associated with the QT segment in ECGs. These common features are referred to as classical biomarkers. We propose a way to automatically extract features from the MEA measurements that are designed to perform well in a classification context. First a set of biomarkers is built. The set is referred to as dictionary and each biomarker is referred to as an entry in the following. Then we define new features, referred to as composite biomarkers, as linear combinations of the dictionary entries. The weights of these linear combinations are found by solving a sparse optimization problem. The optimization procedure uses a data set which consists of experimental MEA measurements, simulated ones or a combination of both.

To predict the effects of drugs onto channel block, we propose to adopt a Machine Learning approach. Machine Learning is a family of statistical methods whose aim is to build predictive models given a (ideally large) data set. There exists a wide variety of such methods: neural networks (Kiranyaz et al., 2016), Support Vector Machine (Hua and Sun, 2001), decision trees (Arikawa et al., 1993), etc. All these methods have proved their performances in many different scenarios of regression and classification, in particular when applied to biological data. In the present work, we propose to use Support Vector Classification (SVC) (Boser et al., 1992) which derives from Support Vector Machine. This method seeks a hyperplane that separates the data samples with a maximum margin. The samples are then classified according to their position with respect to the separating hyperplane.

The paper is organized as follows. First, the methods are described. The *in silico* model is presented and the generation of synthetic data is explained. The algorithm that computes the composite biomarkers is described and the classification tools are presented. Second, the performance of the composite biomarkers and of the classification tools are studied in different drug classification scenarios. The composite biomarkers are compared to the classical ones using two different classification strategies. Finally, composite biomarkers computed with experimental data only and with a mixed set of experimental and synthetic data are compared.

## Methods

### Equations

#### Bidomain equations and ionic model

Let Ω be the domain representing a MEA's well. The thickness of the layer of cells being small compared to the size of the well, the problem is assumed to be two-dimensional. We denote by *A*_m_, *C*_m_ the surface area of membrane per unit volume of tissue, the membrane capacitance, and the thickness of the cell layer, respectively. The intra and extra-cellular conductivity tensors σ_i_ and σ_e_ are assumed to be scalar. The parameters values are reported in Table 1. The propagation of the transmembrane potential *V*_m_ and the extracellular potential ϕ_e_ are modeled in Ω with the bidomain model (Tung, 1978):

(1) $$\left\{ \begin{array}{l} A_{\text{m}}C_{\text{m}}\frac{\partial V_{\text{m}}}{\partial t}+A_{\text{m}}I_{\text{ion}}(V_{m},w)-\nabla \cdot (\sigma_{\text{i}}\nabla V_{\text{m}})-\nabla \cdot (\sigma_{\text{i}}\nabla \phi_{\text{e}})=A_{\text{m}}I_{\text{app}},\\ -\nabla \cdot ((\sigma_{\text{i}}+\sigma_{\text{e}})\nabla \phi_{\text{e}})-\nabla \cdot (\sigma_{\text{i}}\nabla V_{\text{m}})=\frac{1}{z_{\text{thick}}}\sum_{e_{k}}\frac{I_{\text{el}}^{k}}{\left|e_{k}\right|}\chi_{e_{k}}. \end{array}\right.$$

**Table 1:** Bidomain model parameters.

***A*_m_**	***C*_m_**	**σ_*i*_**	**σ_*e*_**	***z*_thick_**
200 cm^−1^	1 μF.cm^−2^	5 mS.cm^−1^	5 mS.cm^−1^	10 μm

**Figure 1:**
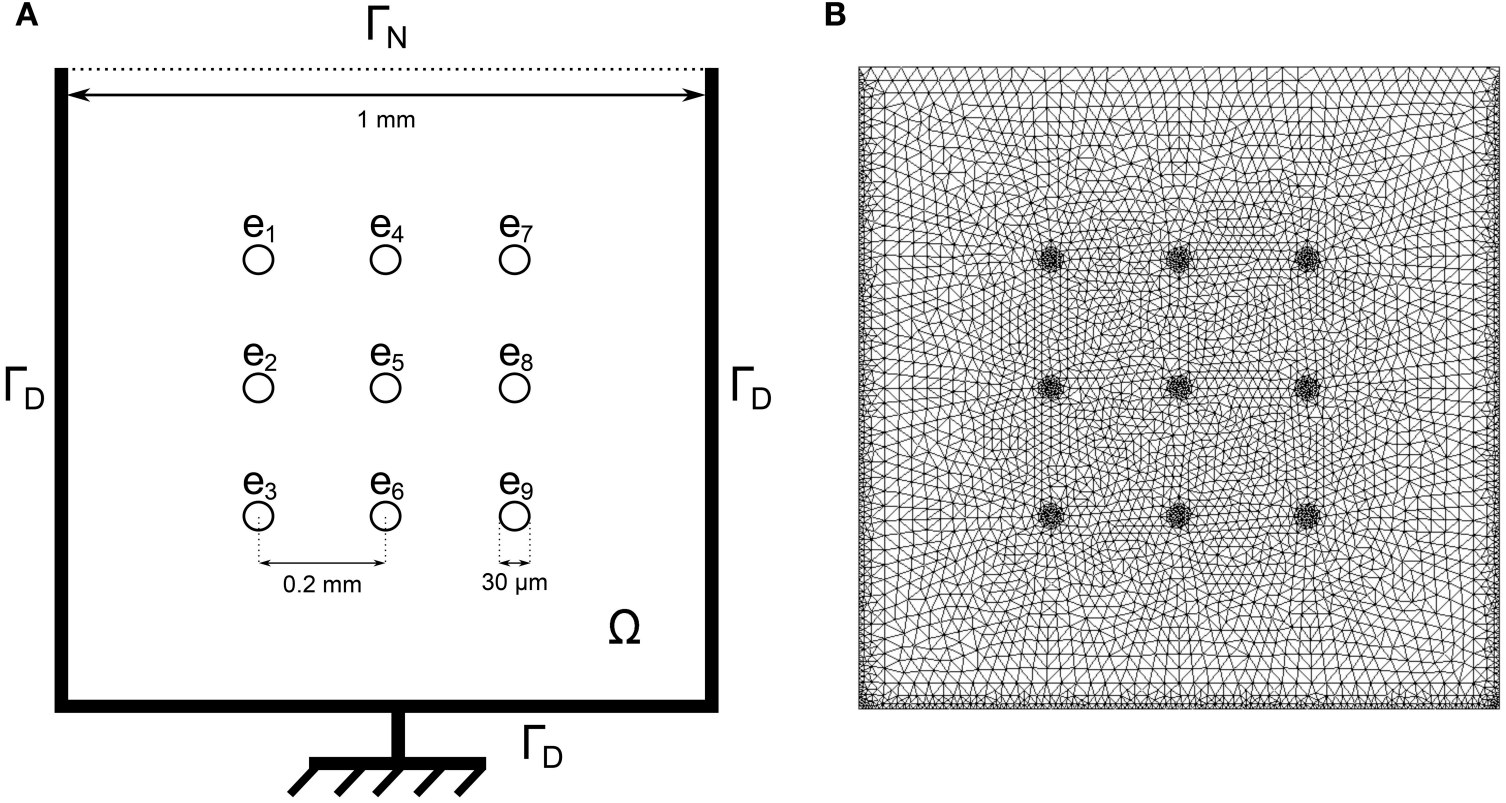
**(A)** Schematic of one well of the nine-electrode MEA device. The bidomain equations are solved in the domain Ω with homogeneous Neumann boundary conditions on Γ_*N*_: $\nabla \phi_{e}\cdot \vec{n}=0$ and homogeneous Dirichlet boundary conditions on Γ_*D*_: ϕ_*e*_ = 0 where the ground is located. **(B)** Corresponding finite element mesh.

In the second equation, $I_{\text{el}}^{k}$ is the electric current which goes through the electrode located at *e*_*k*_, |*e*_*k*_| is the electrode surface and χ_*e*_*k*__ is the characteristic function of *e*_*k*_ (which takes the value 1 on the electrode and 0 elsewhere). An imperfect model for the electrode is used to compute $I_{\text{el}}^{k}$ and described in the Supplementary Material. The activation is assumed to be triggered by a current *I*_app_ that is applied in an arbitrary region of the well with a cycle length of 1,200 ms. The locations of the stimulations are randomized to model the uncertainties of the spontaneous stimulus locations in *in vitro* measurements. This is further explained in the Heterogeneity modeling subsection. The computational domain Ω corresponds to one well of the MEA device as shown in Figure 1. Let **n** be the outward normal to the boundary of the domain Ω. Equations (1) are completed with the following boundary conditions: σ_i_ ∇ ϕ_i_ · **n** = 0 (where ϕ_i_ = *V*_m_ + ϕ_e_), and either ϕ_*e*_ = 0 on the region connected to the ground or σ_e_ ∇ ϕ_e_ · **n** = 0 elsewhere. The ground location is indicated in Figure 1.

The transmembrane ionic current *I*_ion_ is described with the Minimal Ventricular (MV) model (Bueno-Orovio et al., 2008) which includes three currents: fast inward (fi), slow inward (si) and outward (so) currents. The reader is referred to the original publication for more details. Schematically, *I*_ion_ depends on *V*_m_ and on gating variables **w** = (*w*_*j*)1≤*j*≤3_, solution of a system of three non-linear ordinary differential equations. A conductance coefficient *g*_*s*_, with *s* = *fi, si* or *so*, controls the activity of the idealized channels associated with each of three currents of the model.

The partial differential equations are discretized in space by means of P1 finite elements, and in time by using backward differentiation formula (BDF) schemes with adaptive time steps and order provided by Sundials' CVODE library (Hindmarsh et al., 2005). The quantity of interest is the extra-cellular potential, also referred to as field potential (FP). It is a function of time and recorded at the electrodes locations.

##### Synthetic measurements

**Figure 2:**
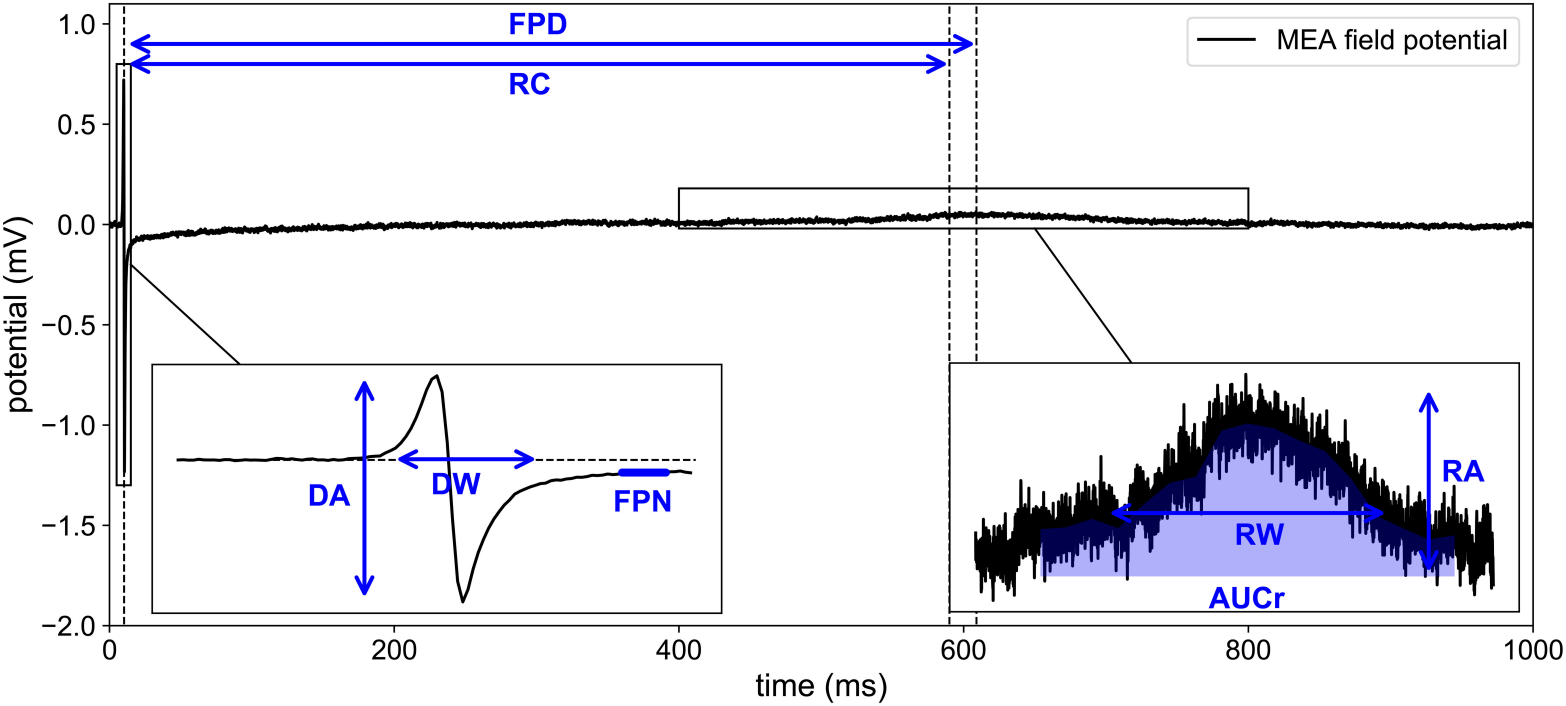
Experimental recording of MEA field potential. Eight biomarkers are extracted from the time series: DA, depolarization amplitude; DW, depolarization width; RA, repolarization amplitude; FPD, field potential duration; AUCr, area under repolarization curve; RC, repolarizarion center; RW, repolarization width; FPN, field potential notch.

**Figure 3:**
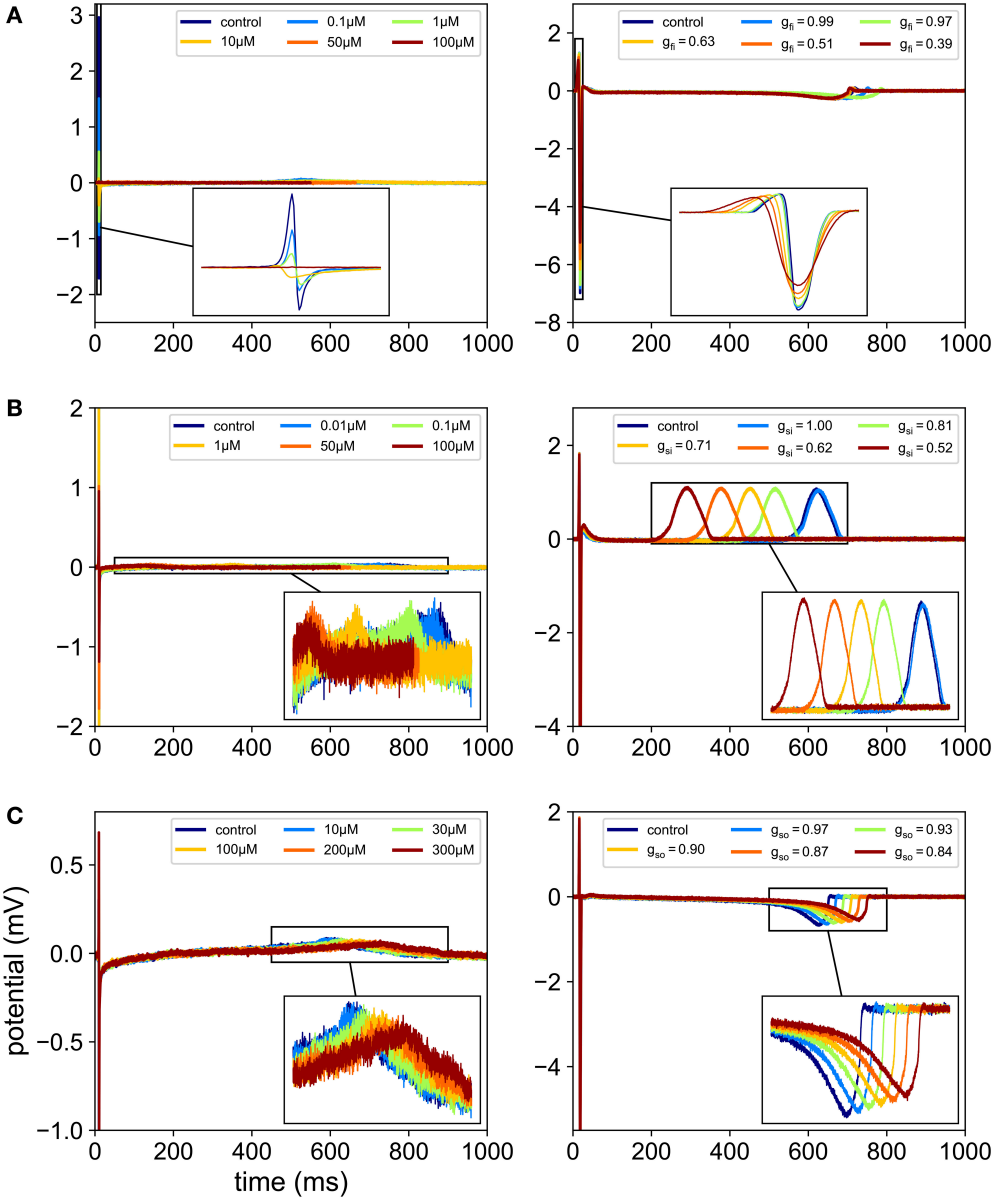
Comparison between *in vitro* and *in silico* MEA FP recordings. In each case, the FPs are recorded in control conditions and for five different drug concentrations. For the *in silico* measurements, the drugs effects are modeled using Equation (2), which amounts to reducing *g*_*fi*_, *g*_*si*_ or *g*_*so*_ depending on the ion channel affected by the drug. **(A)** Effect of flecainide (sodium antagonist drug) on experimental recordings (left) and effect of a virtual sodium antagonist drug on simulated MEA FPs (right). **(B)** Effect of diltiazem (assumed to be mainly calcium antagonist in this study) on experimental recordings (left) and effect of a virtual calcium antagonist drug on simulated MEA FPs (right). **(C)** Effect of moxifloxacin (potassium antagonist drug) on experimental recordings (left) and effect of a virtual potassium antagonist drug on simulated MEA FPs (right).

In the present work, the computational model is used to generate synthetic MEA measurements. The main idea is to enrich the experimental data set with *in silico* measurements to make the classification more robust, in particular by exploring regions of the parametric space that are not covered by the experience. For a given set of conductances, the model is evaluated and the electrodes FPs are recorded. The conductances are chosen as to represent meaningful scenarios, as explained later in the Results section. To mimic experimental measurements, a zero-mean Gaussian noise of standard deviation 10 μV is added to the FPs (see Figure 2). A heterogeneity model of some ionic parameters is also considered to replicate the variability exhibited by the experimental measurements. This model is described later in this section. The stimulation location is also varied to model the uncertainty of the spontaneous stimulus location in the experiments. Figure 3 shows examples of synthetic recordings generated using the aforementioned *in silico* model. The FPs are simulated for three different scenarios. The scenarios consist in simulating the effects of sodium, calcium and potassium antagonist drugs, in each case with five different concentrations. In Supplementary Figure 1 a simulated FP recorded on an electrode is shown with the simulated action potential recorded on the same electrode.

##### Steady-state regime

**Figure 4:**
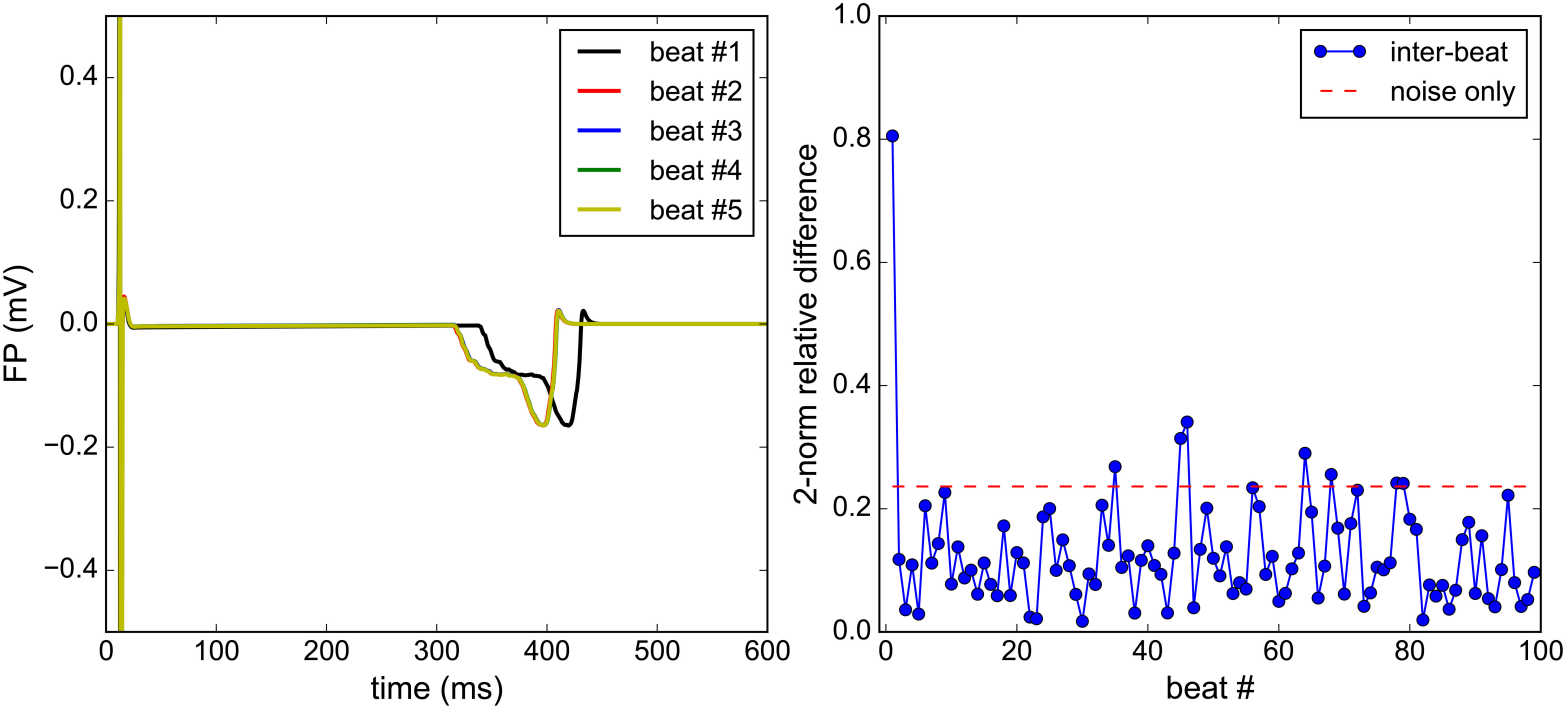
Steady-state analysis: the Bidomain equations are solved for 100 consecutive beats. Qualitatively, a satisfactory steady state is reached at the second beat **(left)**. The beat-to-beat relative difference of the FP is monitored (right) and is to be compared to the relative difference between two identical solutions, each polluted by an independent noise **(right)**.

Because the initial conditions of the ionic model do not correspond to those of a steady-state regime, several beats may need to be simulated before reaching a regime where there is negligible beat-to-beat variations. A numerical experiment was carried out to determine when this regime is reached. Figure 4 shows superimposed consecutive simulated FPs and the normalized beat-to-beat variations in the FP. When considering noisy synthetic measurements as described above, the steady-state is assumed to be reached when the beat-to-beat variations are comparable to variations induced by noise only. The beat-to-beat variability observed after this beat may be imputed to the coarseness of the mesh, the time discretization errors and the fluctuations of the ionic model itself. In the present work, the steady-state is assumed to be reached at the second beat. Therefore, the simulations are run for two cardiac cycles and the second beat is recorded to be used as a synthetic measurement.

#### Drug modeling

We chose to model the action of drugs on the ion channels by the conductance-block formulation of the pore block model (Bottino et al., 2006; Mirams et al., 2011; Zemzemi et al., 2013). This simple approach, which relies on a small number of parameters, was shown in Abbate et al. (2017) to be able to reproduce the expected effects of several drugs on MEA signals. The conductance of a given channel *s* is given by:

(2) $$g_{s}=g_{control,s}{\left[1+{\left(\frac{\left[D\right]}{{\text{IC}}_{50}}\right)}^{n}\right]}^{-1},$$

where *g*_*control,s*_ is the drug-free maximal conductance, [*D*] is the drug concentration, IC_50_ is the value of the drug concentration at which the peak current is reduced of 50%, *n* is the Hill coefficient. In this work, *n* will be assumed to be equal to 1.

#### Heterogeneity modeling

**Figure 5:**
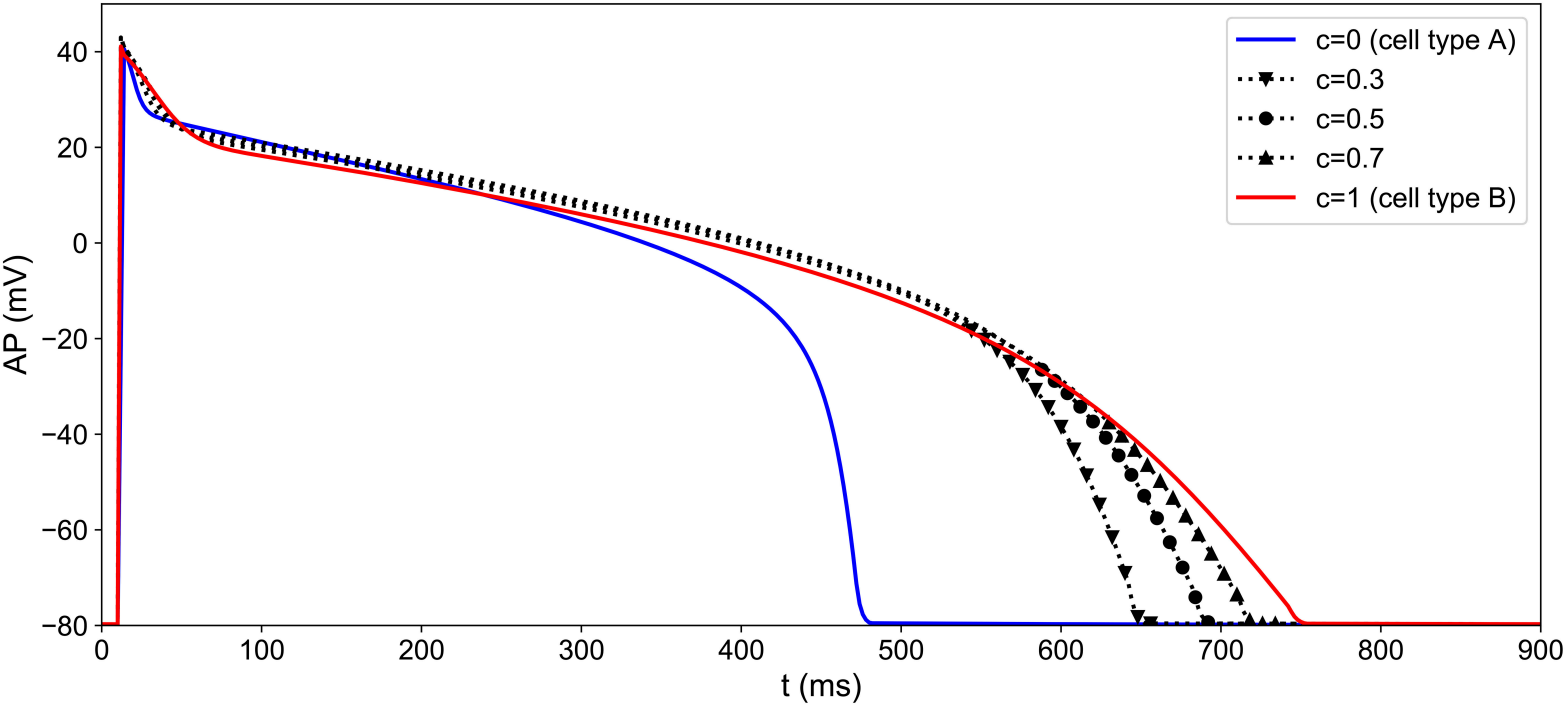
Heterogeneity modeling: different APs obtained by simulating the MV model with different values of the heterogeneity parameter *c*. The heterogeneity parameter is a function of space and its pattern differs from one well to another (see Figure 6).

A typical experimental MEA FP measurement exhibits both a depolarization spike and a repolarization wave (see Figure 2). Using the computational model described above, the repolarization wave is usually too small compared to what is observed in experiments. As noted in Abbate et al. (2017), the repolarization wave provided by this model is larger when the domain includes cells with different APDs. In Abbate et al. (2017), the cell heterogeneity was defined on a checkerboard arbitrarily chosen in the MEA's well. We propose here a different approach, based on a probabilistic description of the heterogeneity. The tissue is supposed to be a continuous mixture of two cell types: A and B. We make the assumption that the transition between these two types can be described by a single space dependent parameter *c*(*x, y*) as follows:

(3) $$p(x,y)=(1-c(x,y))p^{\left(A\right)}+c(x,y)p^{\left(B\right)},$$

where *c* is a random process with values in [0, 1] and **p**^(*A*)^ (resp. **p**^(*B*)^) the set of 19 parameters of the MV model corresponding to cell type A (resp. B). The values of **p**^(*A*)^ and **p**^(*B*)^ are given in Supplementary Table 1. The APs corresponding to different realizations of *c* are shown in Figure 5. We make the hypothesis that the spatial variations of *c* are structured by a normal correlation function *f*_*c*_:

(4) $$f_{c}\left[\left(\begin{array}{c} x\\ y \end{array}\right),\left(\begin{array}{c} x^{\prime }\\ y^{\prime } \end{array}\right)\right]=\exp[-\frac{{\left(x-x^{\prime }\right)}^{2}+{\left(y-y^{\prime }\right)}^{2}}{2l_{c}^{2}}],$$

where *l*_*c*_ is the correlation length, set to *l*_*c*_ = 0.25 mm in the present work. To discretize the random process *c*, we compute the correlation matrix on the finite element mesh used for the discretization of the bidomain equations. The correlation matrix $\text{C}=\left[C_{i,j}\right]\in {\mathbb{R}}^{N_{\text{mesh}}\times N_{\text{mesh}}}$ reads:

(5) $$C_{i,j}=f_{c}\left[\left(\begin{array}{c} {\hat{x}}_{i}\\ {\hat{y}}_{i} \end{array}\right),\left(\begin{array}{c} {\hat{x}}_{j}\\ {\hat{y}}_{j} \end{array}\right)\right],$$

where *N*_mesh_ is the total number of mesh nodes and $\left({\hat{x}}_{i},{ŷ}_{i}\right)$ are the coordinates of the *i*th node. The eigenpairs of **C** are denoted by (λ_*i*_, Φ_*i*_), and ordered by decreasing order of the eigenvalues λ_*i*_. By a convenient abuse of notation, we denote by $\left(\hat{x},ŷ\right)\to \Phi_{i}\left(\hat{x},ŷ\right)$ the function of the finite element space associated with the eigenmode Φ_*i*_. Finally, the discretized heterogeneity field is approximated by the following truncated expansion:

(6) $$c(\hat{x},\hat{y},\xi )=\sum_{i=1}^{n_{c}}\xi_{i}\Phi_{i}(\hat{x},\hat{y})$$

where ξ = (ξ_*i*_)*i* = 1…*n*_*c*_ is a random vector and *n*_*c*_ a truncation index chosen so that the truncation explains at least 99% of the variance. In other words, *n*_*c*_ is the smallest index *n* such that the following criterion is verified:

(7) $$\frac{\sum_{i=1}^{n}\lambda_{i}}{\sum_{i=1}^{N_{\text{mesh}}}\lambda_{i}}>0.99\;.$$

**Figure 6:**
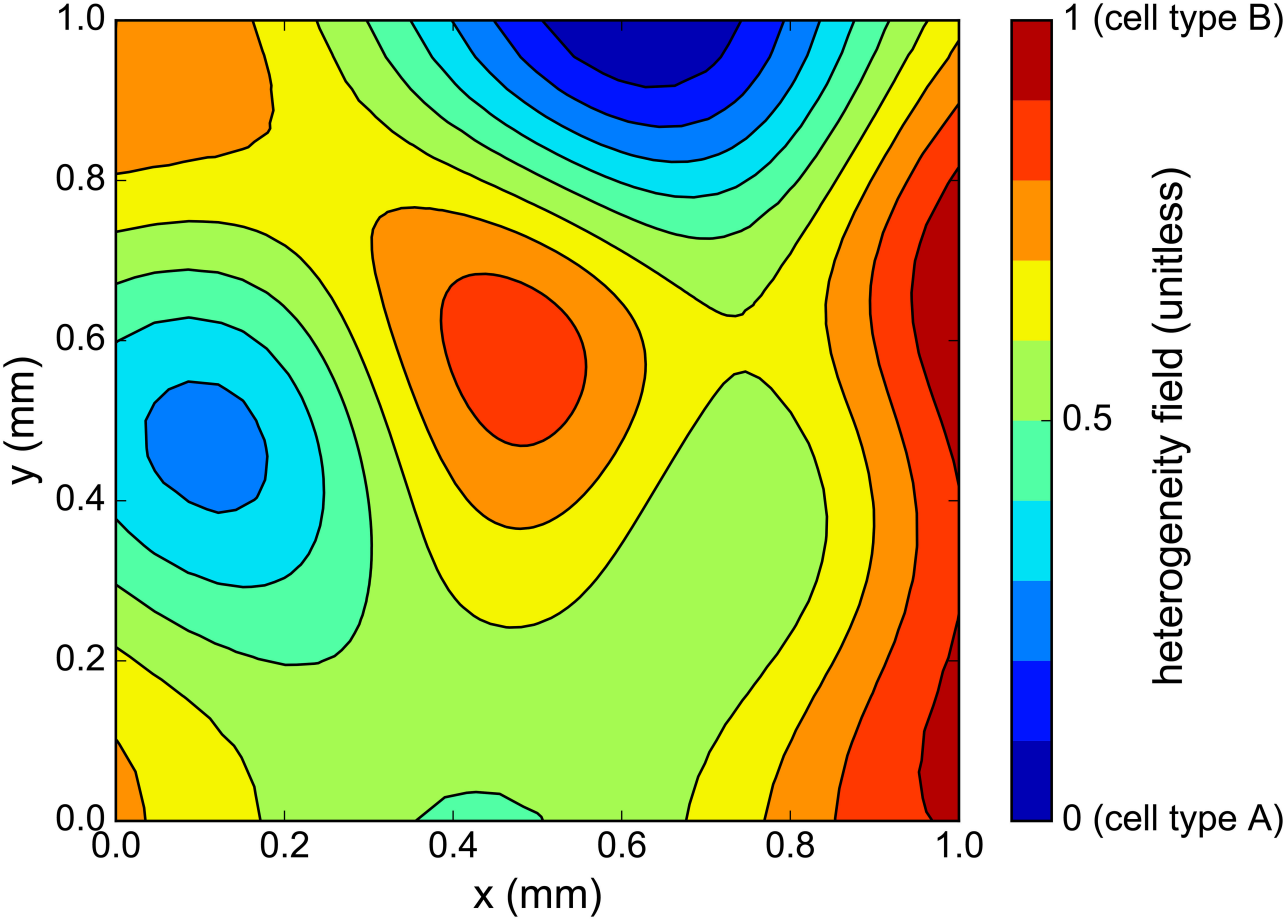
One sample of cell heterogeneity field *c*(*x, y*) generated using the correlation matrix method. As *c* ranges from 0 to 1, the cell action potential varies from that of cell type “A” to cell type “B” (see Figure 5).

In our case, the choice of *l*_*c*_ and the domain geometry yields *n*_*c*_ = 14. Heterogeneity fields can now be generated simply by sampling the random variable ξ. In the present work, *N*_*h*_ = 128 heterogeneity fields were generated by sampling ξ from an uncorrelated uniform distribution over ${\left[-1,1\right]}^{n_{c}}$, and each sample is rescaled to range between 0 and 1. An example of heterogeneity field is presented in Figure 6.

The observed variations in the experimental MEA FP recordings are also attributable to fluctuations in the stimulation location. In practice, the hiPSC-CM are not electrically stimulated: a stimulus arises spontaneously in the medium, probably due to the presence of pacemaker cells. The location of the spontaneous stimulation is not known to the experimentalist. We make the assumption that the location is random and therefore model it with a random uniform law over the square [0.15, 0.85]^2^ where Ω = [0, 1]^2^ is the complete domain.

To conclude, in a given experimental setting, we know neither the stimulation position nor the cell distribution inside the well and we would like the classification method to be robust with respect to all these unknown, random elements. This is why, when generating synthetic MEA FPs using our *in silico* model, we introduce two sources of uncertainty: the heterogeneous CM field and the stimulation location.

### Biomarkers

Biomarkers may be defined as quantities extracted from a signal that convey information about hidden quantities of interest. In our case, the biomarkers are features extracted from the MEA FP which would ideally provide information about the conductances of interest: *g*_*fi*_, *g*_*so*_, *g*_*si*_. In this section, we present different choices of biomarkers to be used in a classification context.

#### “Classical” biomarkers

The MEA FP can be split into two regions of interest: the depolarization and the repolarization. The depolarization observed at one electrode corresponds to the local depolarization of the cardiomyocytes. The depolarization amplitude (DA, referred to as spike amplitude in Clements and Thomas, 2014) may be qualitatively linked to the AP upstroke velocity. This biomarker is commonly associated with the activity of the fast sodium channel (*g*_*fi*_ for the MV model). The repolarization amplitude (RA) may be qualitatively linked to some extent to the AP repolarization slope and to a bigger extent to spatial heterogeneities in AP durations. Once the depolarization and repolarization have been detected, it is possible to measure the FP duration (FPD), simply as the difference between the repolarization and depolarization times. The FPD is a commonly used biomarker (Navarrete et al., 2013; Clements and Thomas, 2014) which may be seen as a surrogate for APD in patch clamp experiments and QT interval in electrocardiograms. Both biomarkers RA and FPD are associated with the activity of the potassium and calcium currents (*g*_*so*_ and *g*_*si*_ in the MV model). As explained above, each (real or numerical) experiment is performed both in drug-block conditions and in control condition. Because of the significant variability of measurements in MEA, it is important to consider the variations observed in the FP in drug block conditions with respect to the control conditions to isolate the effect of the drug from other sources of variability: tissue variability, stimulation protocol, etc. Therefore, as proposed in Raphel et al. (2017), the features of interest are the biomarkers in drug block condition divided by the biomarkers in control conditions. For instance, the depolarization amplitude is actually the following ratio:

(8) $${\text{DA}}_{\text{ratio}}=\frac{{\text{DA}}_{\text{drug}}}{{\text{DA}}_{\text{control}}}$$

For the sake of clarity in the notation, the subscript “ratio” is omitted in the following and any biomarker actually refers to a ratio with the control value. For each MEA measurement, the FP is recorded at each of the nine electrodes. Again, the important variability in the measurements motivates the use of robust features. Since the behavior of the FP may greatly vary from one electrode to another, the median of the biomarkers over all electrodes is in practice a good choice of features. In the following, the set of biomarkers $\left\{\tilde{\text{DA}},\tilde{\text{RA}},\tilde{\text{FPD}}\right\}$ is referred to as the classical biomarkers, where the ˜ operator denotes the median over all nine electrodes.

#### Composite biomarkers

The rationale behind the choice of biomarkers described above is only qualitative and oftentimes does not represent the best set of features in a classification context. Here, we adopt a more automatic strategy to select the best set of biomarkers for a given experimental scenario. First, the set of features to be extracted from a given FP is enriched with other features.

It is indeed possible to extract additional quantities from the FP other than DA, RA, and FPD. We propose to compute also, for each electrode of the MEA, the following features: the area under curve of the repolarization wave (AUCr), the repolarization center (RC), the repolarization width (RW), the FP notch (FPN), and the depolarization width (DW). The details on how to compute these additional biomarkers are described in Appendix A (Supplementary Material) and illustrated in Figure 2. Ratios of these quantities are also added to the dictionary of features: RA/DA, DA/RA, RA/FPD, FPD/RA, DA/FPD, FPD/DA, RA/RW, RW/RA. Each feature is actually a ratio with its control counterpart as described in Equation (8). To include the information of all nine electrodes, the median (denoted by the ˜ operator), mean (denoted by the <> operator) and maximum values (denoted by a max subscript) over the electrodes are retained in the dictionary. We finally add the conduction velocity (CV) which is not an electrode-wise quantity but defined using all nine electrodes signals as explained in Appendix A (Supplementary Material). This amounts to a total of *N*_*b*_ = 41 features reported in Table 2. The extended set of features is referred to as the dictionary or the biomarkers dictionary. Each biomarker is referred to as an entry, denoted by *b*_*j*_, 1 ≤ *j* ≤ *N*_*b*_, in the following.

**Table 2:** Indices of the biomarkers dictionary entries.

**Index (median)**	**Index (mean)**	**Index (max)**	**Entry**
0	8		DA
1	9		RA
2	10		FPD
3	11		AUC_r_
4	12		RC
5	13		RW
6	14		FPN
7	15		DW
16	24	32	RA/DA
17	25	33	DA/RA
18	26	34	RA/FPD
19	27	35	FPD/RA
20	28	36	DA/FPD
21	29	37	FPD/DA
22	30	38	RA/RW
23	31	39	RW/RA
	40		CV

**Figure 7:**
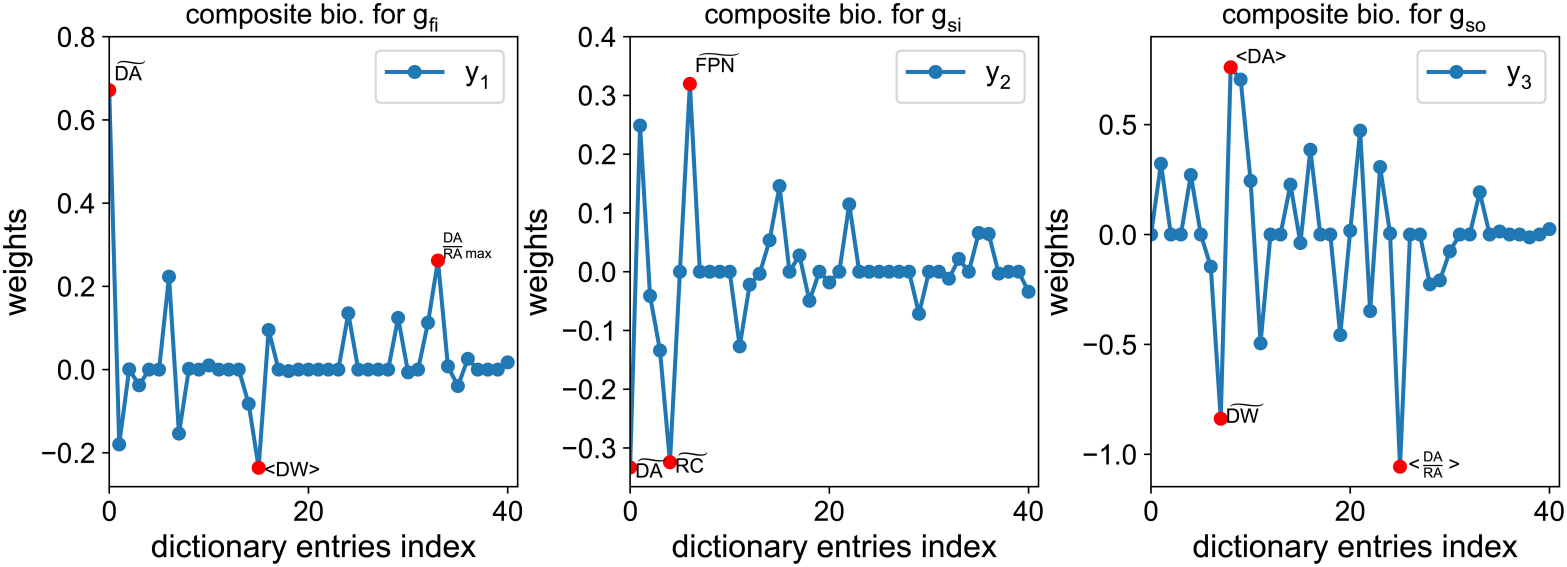
Example of composite biomarkers weights. The three highest weights (in absolute value) are highlighted by a red dot for each composite biomarker. Note that some classical biomarkers are selected by the method: $\tilde{\text{DA}}$ for *g*_*fi*_, $\tilde{\text{RC}}$ (closely related to the FPD) for *g*_*si*_ and RA in the ratio (DA/RA) for *g*_*so*_.

Before going into further details about the numerical methods, let us now explain the purpose of the composite biomarkers. The purpose of the method is to associate each conductance *g*_*fi*_, *g*_*si*_, *g*_*so*_ with a composite biomarker that is maximally correlated with it and minimally correlated with the others. For instance, the composite biomarker, denoted by *y*_1_, associated with *g*_*fi*_ is maximally correlated with *g*_*fi*_ while being minimally correlated with *g*_*si*_ and *g*_*so*_. The main idea is that by observing *y*_1_ we have good information about the hidden variations of *g*_*fi*_ which is not tampered by simultaneous variations of *g*_*si*_ or *g*_*so*_. The composite biomarkers are defined as weighted linear combinations of the dictionary entries. We also require that the weights are sparse, meaning there are a lot of zero weights. This makes the composite biomarkers more easily interpretable. Indeed, they can be seen as a combination of only a small subset of the dictionary entries, ideally including the classical biomarkers as seen in Figure 7.

The weights of such a combination are solution of an optimization problem. First, let us introduce some notation. We denote by *y*_1_ (resp. *y*_2_, *y*_3_) the composite biomarker (to be determined) associated with *g*_*fi*_ (resp. *g*_*si*_, *g*_*so*_). From now on, the conductances (*g*_*fi*_, *g*_*si*_, *g*_*so*_) are denoted by **θ** = (θ_1_, θ_2_, θ_3_). Each dictionary entry is considered as a function of **θ**. The composite biomarkers are sought as a linear combination of the dictionary entries:

(9) $$y_{h}(\theta )=\sum_{j=1}^{N_{b}}w_{j}^{\left(h\right)}b_{j}(\theta ),\;1\leq h\leq 3,$$

where the weights ${\text{w}}^{\left(h\right)}=\left(w_{j}^{\left(h\right)}\right)\in {\mathbb{R}}^{N_{b}}$ are the unknowns of the problem. These weights are sought so that *y*_*h*_(**θ**) is maximally correlated with θ_*h*_ and minimally correlated with θ_*k*_, ∀*k* ≠ *h*. This may be stated as follows:

(10a,10b,10c) $$\forall h\in \{1,\ldots ,3\},\;\{\begin{array}{ccc} \underset{y_{h}}{\max} & \text{cov }(y_{h}(\theta ),\theta_{h}) & \;\\ \underset{y_{h}}{\min} & |\text{cov }(y_{h}(\theta ),\theta_{k})|, & \forall k\neq h\\ \text{s}.\text{t}. & \text{var }(y_{h}(\theta ))=1 & \; \end{array}$$

where cov(·, ·) and var(·) are respectively the covariance and variance operators. In the following, we assume that each component of **θ** is a zero-mean unit-variance random variable. This is achieved in practice by a simple rescaling of the conductances samples. We also adopt the following notation:

(11) $${\tilde{b}}_{j}(\theta )=b_{j}(\theta )-𝔼[b_{j}(\theta )],$$

where 𝔼[·] is the expectation operator. The problem may now be recast into an optimization problem where the cost function to be minimized reads:

(12) $$J(w^{\left(h\right)})=J_{C}(w^{\left(h\right)})+J_{N}(w^{\left(h\right)})+J_{P}(w^{\left(h\right)}),$$

where

(13a) $$J_{C}(w^{\left(h\right)})=\frac{1}{2}||Cw^{\left(h\right)}-e^{\left(h\right)}|{|}^{2}\text{ where }C_{kj}:=𝔼(\theta_{k}{\tilde{b}}_{j}),\;e_{k}^{\left(h\right)}:=\delta_{kh},$$

(13b) $$J_{N}(w^{\left(h\right)})=\frac{\xi }{2}{\left(w^{(h)T}Gw^{\left(h\right)}-1\right)}^{2}\text{ where }G_{ij}:=𝔼({\tilde{b}}_{i}{\tilde{b}}_{j}),$$

(13c) $$J_{P}(w^{\left(h\right)})=\frac{\lambda_{h}}{N_{b}}||w^{\left(h\right)}|{|}_{1}.$$

Let us now explain each term of Equation (14). $J_{C}\left(w^{\left(h\right)}\right)$ corresponds to Equations (10a,b). It measures the discrepancy to the ideal situation where cov(*y*_*h*_(**θ**), θ_*h*_) = 1 and cov(*y*_*h*_(**θ**), θ_*k*_) = 0, ∀*k* ≠ *h*.

$J_{N}\left(w^{\left(h\right)}\right)$ is a relaxation of the constraint in Equation (10a). ξ is a regularization parameter that is set to 1 in practice.

$J_{P}\left(w^{\left(h\right)}\right)$ is a regularization term by penalization of the 1–norm of ***w***^(*h*)^, where λ_*h*_, 1 ≤ *h* ≤ 3, are regularization parameters. ℓ_1_ penalized cost functions tend to promote sparse solutions (Tibshirani, 1996). Sparse solutions for ***w***^(*h*)^ are interesting in that they offer a more interpretable decomposition onto the dictionary entries (since most weights are zero) than what an ℓ_2_ penalization would yield.

We now discretize the problem by considering *N* samples of the parameters **θ** drawn over a parameter space Θ ⊂ ℝ^3^. The expectation operator is approximated using a quasi-Monte-Carlo quadrature rule and the cost function in Equation (12) is minimized using a Nesterov accelerated gradient descent (O'Donoghue and Candes, 2015). The Monte-Carlo samples may come from synthetic or experimental measurements. For synthetic measurements, the conductances are known, but this is not the case for experimental measurements. In that case, an approximation of these conductances is computed using Equation (2). Note that the solution weights depend strongly on the choice of samples used for the Monte-Carlo approximations.

**Figure 8:**
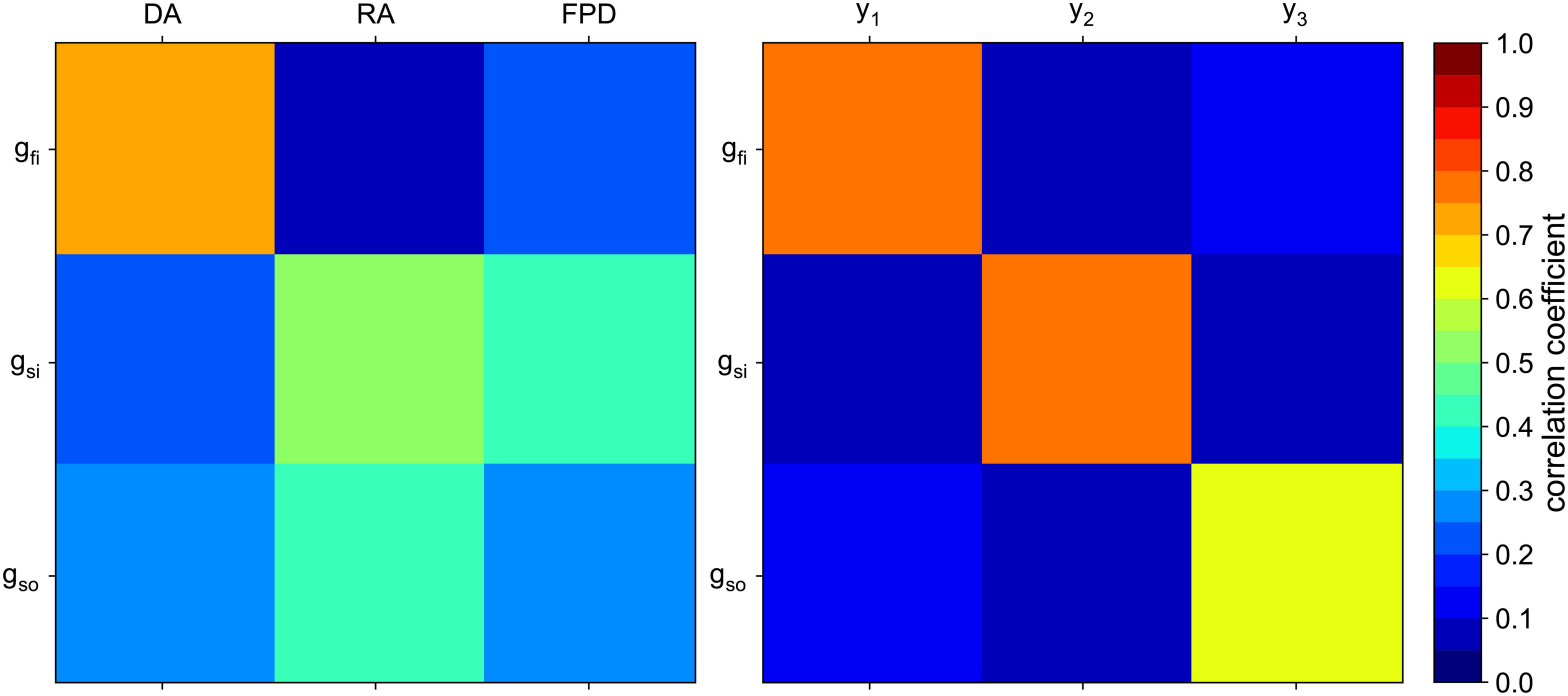
Correlation matrix of the conductances of interest with the “classical” biomarkers **(Left)** and with the composite biomarkers **(Right)**.

An example of the obtained weights is shown in Figure 7. Interestingly, the classical biomarkers are still among the most weighted features. The correlation between the conductances of interest and the composite biomarkers is compared to the correlation with the classical biomarkers in Figure 8. The correlation between two quantities *u* and *v* is defined as follows:

(14) $$\text{cor}(u,v)=\frac{\text{cov}(u,v)}{\sqrt{\text{var}(u)\text{ var}(v)}}.$$

As expected, each composite biomarker is well correlated with its associated conductance whereas uncorrelated with the others. This is not the case for the classical biomarkers. The results in the next section show that such a choice of features improves the classification performance.

### Experimental data set

The MEA considered in the present work is a 6-well MEA with nine electrodes per well. Its geometry as well as the corresponding finite element mesh is shown in Figure 1. The MEA measurements come in the form of FP recordings corresponding to the different electrodes of the different wells of the MEA. These recordings come in the form of time series where several cardiac cycles, or beats, are recorded. The time resolution of the MEA recordings is 10 kHz. We extracted several beats on each electrode from each well of the MEA. Data were provided by Janssen Pharmaceutica NV using MC_Rack (Multi Channel Systems GmbH) and post-processed by NOTOCORD Systems (NOTOCORD-FPS 3.0 software). The hiPSC-CM used in this study are a commercially available line of cells (iCell Cardiomyocytes) and were provided by the CDI (Cellular Dynamics International) company.

After thawing, the hiPSC-CM were precultivated for 7 days before being plated on the MEA. Then the cells were cultivated again from 6 to 7 days. Prior to the experiments, the cells rested for 15 min inside the MEA. The recordings come in series of 2 min each and a wash-in period of 5 min was allocated before changing compound concentrations. Up to two different hiPSC-CM cultures were used and each experiment was repeated from 5 to 12 times.

As explained earlier the recordings were made in control conditions (no drug) and with different drugs at different concentrations levels. Figure 3 shows examples of experimental recordings in control conditions and with five different concentrations of flecainide, diltiazem and moxifloxacin. The drugs used for the present study are summarized in Table 3. The corresponding concentrations are presented in Table 4. The IC_50_ values that were used in the study are also reported and are in the range of those reported in Crumb et al. (2016). Note that the diltiazem was recorded in two different wells (A and B) since it was the only calcium-antagonist drug in the experimental data that were made available to the authors. The experimental process consists in adding five times a compound at increasing concentrations in a given well. Thus, including the control condition record, we finally obtain field potentials for six contexts in each well. Equation (2) was used to obtain an approximation of the conductances values associated with the experimental measurements which are needed for the composite biomarkers calculations. The Hill coefficients and IC_50_ values are given in the Supplementary Material of Kramer et al. (2013) and Mirams et al. (2011). Concerning the dictionary of features, a few adjustments need to be made in some cases. Indeed, it appears that at some high concentration levels of mexiletine, there is simply no action potential (because the sodium channels are too blocked) and therefore the field potential is a flat line. To take this into account, the values of dictionary entries are set to the ones at the last concentration where an action potential was observed. In addition, all features where DA is in the numerator position in a ratio are set to zero for this concentration.

**Table 3:** Repartition of the available (experimental and synthetic) data set.

**Drug name**	**Blocked ionic channel**	**Associated conductance**	**ID**	**SVC class label**	**# Experiments**
Mexiletine	Sodium	*g*_*fi*_	0	0	160
Flecainide	Sodium	*g*_*fi*_	1	0	120
Diltiazem A	Calcium	*g*_*si*_	2	1	160
Diltiazem B	Calcium	*g*_*si*_	3	1	160
Moxifloxacin	Potassium	*g*_*so*_	4	2	120
Dofetilide	Potassium	*g*_*so*_	5	2	160
Synth. A	Sodium	*g*_*fi*_	6	0	155
Synth. B	Calcium	*g*_*si*_	7	1	155
Synth. C	Potassium	*g*_*so*_	8	2	155

*The ID is used in the data set splitting (see Table 5). The SVC class label corresponds to the associated blocked channel conductance*.

**Table 4:** Summary of the drugs information constituting the experimental measurement set.

**Concentration index**	**Mexiletine**	**Flecainide**	**Diltiazem**	**Moxifloxacin**	**Dofetilide**
1	0.01 μM	0.1 μM	0.01 μM	10.0 μM	0.1 nM
2	0.1 μM	1.0 μM	0.1 μM	30.0 μM	1.0 nM
3	1.0 μM	10.0 μM	1.0 μM	100.0 μM	10.0 nM
4	10.0 μM	50.0 μM	5.0 μM	200.0 μM	50.0 nM
5	50.0 μM	100.0 μM	10.0 μM	300.0 μM	100.0 nM
Main channel blocked	Sodium	Sodium	Calcium	Potassium	Potassium
IC_50_	43.0 μM	6.2 μM	0.75(or0.45) μM	86.2 μM	30.0(or5.0) nM

*Five concentrations were studied for each drug. The IC_50_ values are reported as well as the main channel blocked by each drug (in the scope of the single channel block assumption)*.

### Classification

#### Support vector classification

Support vector classification (Boser et al., 1992) (SVC) is an adaptation of the support vector machine (SVM) method in a classification setting. Classification generally consists in attributing labels to inputs. The available data set, comprising both inputs and labels, is generally split into a training set used to build the classifier and a validation set to test the classifier. The inputs are often multidimensional and in our case correspond to the biomarkers, whether classical or composite. The labels are integers that represent the classes to which the inputs are assigned. These classes are mutually exclusive, meaning one sample can only belong to a single class. SVC belongs to the so-called supervised methods since the labels are known, at least for the training set. The main idea behind SVC is to maximize the margin between the inputs and the decision boundary (Boser et al., 1992). In the linear case, the decision boundary is a hyperplane of the input space. In general, however, this is not sufficient to properly separate the samples according to their classes. A common way to obtain more complex boundary decisions is to use a so-called “kernel trick” (Schölkopf and Smola, 2002) which is based on a mapping from the input space to a higher-dimensional space where the existence of a separating hyperplane is more likely. In the present case, the labels are “sodium antagonist,” “calcium antagonist,” and “potassium antagonist,” respectively associated with labels 0, 1, and 2. Among various possible choices of kernels, a Gaussian kernel is employed in this work.

We used a Python implementation of SVC through the Scikit-learn (Pedregosa et al., 2011) machine learning library which itself uses the LIBSVM library (Chang and Lin, 2011). For a given training set, a so-called classifier is built. The classifier is then called to predict the labels of the validation set samples. The predictions are finally compared to the true labels. There exist several metrics to quantify the prediction quality. Two different metrics are considered here: the Cohen's kappa and the receiver operating characteristic area under curve (AUC). The Cohen's kappa is a single scalar designed to measure the performance of multi-class classifiers. Its value ranges from −1 (worst possible classifier) to 1 (perfect classifier), 0 corresponding to a coin-flip classifier. The AUC is defined for each class and measures how a classifier performs with respect to a given class. Its value ranges from 0 (worst) to 1 (best), 0.5 being a coin-flip. Because the classification is repeated several times with different data set splittings, the classification metrics are summarized using their means and standard deviations. The “averaged AUC” corresponds to the average of all AUCs (one AUC per class).

Both metrics are described in detail in the Supplementary Material. We now present two different strategies to employ SVC in the context of drug classification.

##### 3-vs.-3 classification

Since there are three distinct classes in the experimental set, those three classes need to be included in the training set, preferably in equal proportions. The strategy of 3-vs.-3 (3v3) classification consists in dividing the experimental set into a training set and validation set that both include samples from the three classes. Each class is divided into two sub-classes. This is naturally done for the sodium and potassium antagonist classes since they are each comprised of data from two different drugs. For the calcium antagonist class, the diltiazem data is artificially split into two drugs “diltiazem A” and “diltiazem B” (see Table 3). Each subclass is associated with an identification number (ID) from 0 to 5. Therefore, there are 8 possible choices for the training and validation set combinations as summarized in Table 5.

**Table 5:** Different possible splittings of the experimental data set.

**Splitting index**	**0**	**1**	**2**	**3**	**4**	**5**	**6**	**7**
Training set IDs	{0, 2, 4}	{0, 2, 5}	{0, 3, 4}	{0, 3, 5}	{1, 2, 4}	{1, 2, 5}	{1, 3, 4}	{1, 3, 5}
Validation set IDs	{1, 3, 5}	{1, 3, 4}	{1, 2, 5}	{1, 2, 4}	{0, 3, 5}	{0, 3, 4}	{0, 2, 5}	{0, 2, 4}

##### One-vs.-all classification

The One-vs.-All (OvA) classification strategy consists in training one classifier for each class. For each class *j*, the training set labels are modified to take the value 1 for samples in class *j* and 0 otherwise and a classifier is trained on this relabeled training set. In other words, the classifier for class *j* is only trained to recognize whether or not a sample belongs to class *j*. For the validation step, the classifiers do not predict a class label but a probability for a given sample to be in their respective class. Each sample of the validation step goes through each of the three classifiers and the predicted class corresponds to the classifier returning the highest probability. The splitting between training and validation sets is done in the same way as in the 3-vs.-3 classification strategy.

## Results

### Comparison between classical and composite biomarkers

Here the performance of the composite biomarkers in a classification context is compared to that of the classical biomarkers for two different classification strategies. The data set is composed of 880 experiments, each counting one control measurement and 5 measurements at different drug concentration levels. For each experiment, the conductances values and FP features are computed as explained in the Methods section and the labels are defined according to Table 3. The classification results are summarized in the following and more detailed results may be found in Supplementary Tables 3, 4. The statistical significance of the potential improvements in the classification scores attributable to the use of composite biomarkers is studied using an analysis of variance (ANOVA) with a significance level of 0.05.

#### 3v3 classification

The performance of the composite biomarkers compared to the classical ones is evaluated using the 3v3 classification strategy. The classification procedure is carried out for each different splitting of the data set as summarized in Table 5. First, the classification inputs are the 3 classical biomarkers for each drug concentration level:

(15) $$\left\{{\tilde{\text{DA}}}_{c1},{\tilde{\text{RA}}}_{c1},{\tilde{\text{FPD}}}_{c1},\ldots ,{\tilde{\text{DA}}}_{c5},{\tilde{\text{RA}}}_{c5},{\tilde{\text{FPD}}}_{c5}\right\},$$

where *c*_*k*_ is the *k*-th concentration level. Then, the classification inputs are the composite biomarkers for each concentration, computed as explained in the Methods section using the classification training set as samples for the Monte-Carlo approximations. The inputs now read:

(16) $$\{y_{1,c1},y_{2,c1},y_{3,c_{1}},\ldots ,y_{1,c5},y_{2,c5},y_{3,c_{5}}\}.$$

In both cases, the inputs are therefore of dimension 15. Note that for each splitting of the data set, new weights for the composite biomarkers are computed. The classification procedure is carried out in both cases and the results are summarized in Table 6. Regardless of the chosen classification score, the results are significantly better using the composite biomarkers as inputs.

**Table 6:** Comparison between classical and composite biomarkers with the 3v3 classification strategy.

**Score**	**Classical biomarkers**		**Composite biomarkers**	
	**Mean**	**Std**.	**Mean**	**Std**.
Cohen's kappa	0.27	0.16	0.56^+,^^*^	0.25
*g*_*fi*_ AUC	0.74	0.15	0.90^+,^^*^	0.09
*g*_*si*_ AUC	0.98	0.01	1.00^+,^^*^	0.00
*g*_*so*_ AUC	0.69	0.04	0.84^+,^^*^	0.04
Averaged AUC	0.80	–	0.92	–

Variation (^+^increase, ^−^decrease) in the classification scores attributable to the composite biomarkers. ANOVA study:

* *significant at the 0.05 probability level*.

#### OvA classification

The same procedure as in the 3v3 case is applied to the OvA strategy. The classification procedure is carried out with both classical and composite biomarkers as inputs and the results are summarized in Table 7. The prediction of slow outward current block is significantly improved using the composite biomarkers as inputs. Furthermore, the results are overall better when using the OvA approach rather than the 3v3 one.

**Table 7:** Comparison between classical and composite biomarkers. Classification scores in the one-vs.-all scenario.

**Score**	**Classical biomarkers**		**Composite biomarkers**	
	**Mean**	**Std**.	**Mean**	**Std**.
Cohen's kappa	0.44	0.24	0.54^+,^^†^	0.24
*g*_*fi*_ AUC	0.83	0.10	0.74^−,^^†^	0.24
*g*_*si*_ AUC	0.89	0.10	0.94^+,^^†^	0.07
*g*_*so*_ AUC	0.77	0.13	0.92^+,^^*^	0.08
Averaged AUC	0.83	–	0.87	–

Variation (^+^increase, ^−^decrease) in the classification scores attributable to the composite biomarkers. ANOVA study:

* *significant at the 0.05 probability level*,

† *non-significant at the 0.05 probability level*.

In the next section, the addition of synthetic measurements in the computation of the composite biomarkers is investigated. To test whether potential improvements are due to the nature of the added data and not to the increase in the size of the data set, the classification framework was applied to a smaller data set of 440 experiments (i.e., half of the previous data set) for which the results are reported in Supplementary Tables 5, 6. The conclusions of this additional study being similar, this suggests that the classification results are weakly impacted by the size of the data set.

### Using combined experimental and synthetic measurements for the composite biomarkers computation

**Figure 9:**
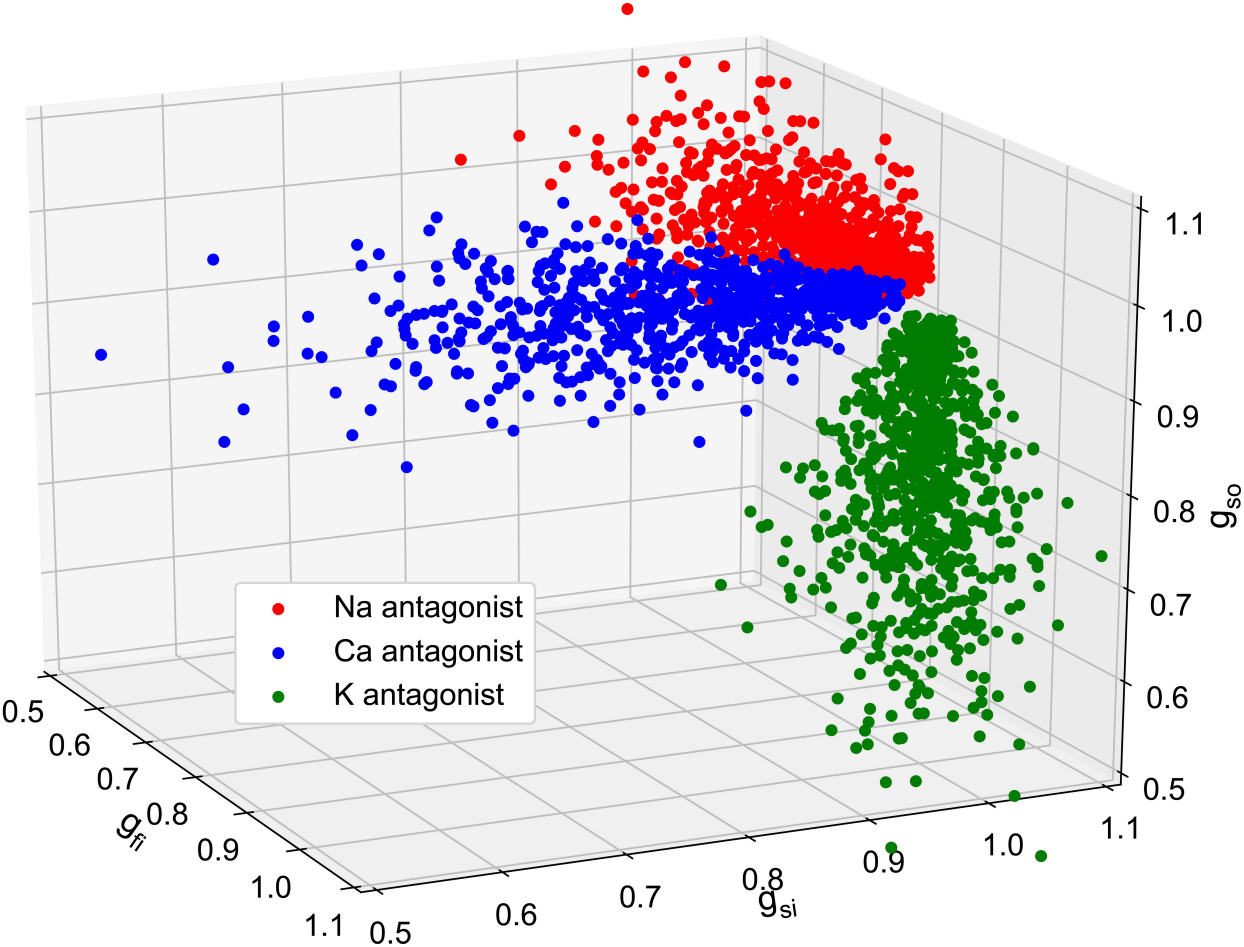
Plot of the 2,325 *in silico* conductances samples. Three populations of 155 virtual drugs were generated according to their ion channel targets: sodium antagonist drugs (red), calcium antagonist (blue) and potassium antagonist (green). For each drug, 5 different concentrations are considered which correspond to 5 different set of conductances. These conductances are then used as inputs to generate *in silico* MEA measurements using the bidomain equations.

Having established that composite biomarkers outperform classical ones in two different classification scenarios, we now investigate the addition of synthetic measurements for the computation of the composite biomarkers weights. To enrich the set of experimental samples used to compute the composite biomarkers, a set of synthetic measurements is built. First, conductances samples are chosen to mimic the effect of drugs as shown in Figure 9. Depending on the most affected conductance, these samples are associated with a synthetic sodium (resp. calcium and potassium) antagonist drug called “synth A” (resp. B and C). 775 samples per drug are chosen which amounts to 155 experiments per drug. and their repartition is summarized in Table 3. This approximately corresponds to a 50/50% split between experimental and synthetic measurements. For each conductance sample, the computational model described in the Methods section is evaluated and the dictionary entries are computed from the simulated FPs. For each experiment, the computational model is also evaluated in control conditions, i.e., with *g*_*fi*_ = *g*_*si*_ = *g*_*so*_ = 1 in order to compute the ratios as defined in Equation (8). The *in silico* measurements are incorporated in the experimental set to create an augmented set. This augmented set is then used to compute the composite biomarkers weights. The same data set splitting procedure as described before is carried out. Note that the synthetic measurements are only used for the composite biomarkers computation and are included neither in the training set nor in the validation set. Again, two classification strategies are explored.

#### Classification results

The classification is carried out using both 3v3 and OvA approaches. The results are summarized in Tables 8, 9 and reported in detail in Supplementary Tables 4, 7. The statistical significance of the modifications in the classification scores standard deviations attributable to the use of synthetic data is assessed using the *F*-test with a significance level of 0.05.

**Table 8:** Comparison between composite biomarkers computed from experiments only and combined experiments and synthetic measurements.

	**Experiments only**		**Experiments** + **synthetic**	
**Score**	**Mean**	**Std**.	**Mean**	**Std**.
Cohen's kappa	0.56	0.25	0.59	0.10^−,^^*^
*g*_*fi*_ AUC	0.90	0.09	0.89	0.06^−,^^†^
*g*_*si*_ AUC	1.00	0.00	1.00	0.00^=^
*g*_*so*_ AUC	0.84	0.04	0.85	0.06^+,^^†^
Averaged AUC	0.92	–	0.91	–

*3v3 classification strategy*.

Variation (^+^increase, ^−^decrease) in the classification scores standard deviations attributable to the use of numerical simulations in the composite biomarkers computation. F-test of variances:

* *significant at the 0.05 probability level*,

† *non-significant at the 0.05 probability level, ^=^no variation*.

**Table 9:** Comparison between composite biomarkers computed from experiments only and combined experiments and synthetic measurements.

	**Experiments only**		**Experiments** + **synthetic**	
**Score**	**Mean**	**Std**.	**Mean**	**Std**.
Cohen's kappa	0.54	0.24	0.63	0.19^−,^^†^
*g*_*fi*_ AUC	0.74	0.24	0.81	0.14^−,^^†^
*g*_*si*_ AUC	0.94	0.07	0.99	0.01^−,^^*^
*g*_*so*_ AUC	0.92	0.08	0.81	0.17^+,^^*^
averaged AUC	0.87	–	0.87	–

*OvA classification strategy*.

Variation (^+^ increase, ^−^ decrease) in the classification scores standard deviations attributable to the use of numerical simulations in the composite biomarkers computation. F-test of variances:

* *significant at the 0.05 probability level*,

† *non-significant at the 0.05 probability level*.

In the 3v3 case, the Cohen's kappa standard deviation is significantly decreased when using the mixed set of experiments and synthetic data. In the OvA case, the standard deviation of the *g*_*si*_ AUC is significantly decreased while that of the *g*_*so*_ AUC is increased.

## Discussion

In this study, a framework for an automatic classification of drugs from MEA measurements has been presented. The framework relies on an *in silico* model of a MEA device, on a feature selection algorithm and on state-of-the-art machine learning tools. The *in silico* model is a PDE model (the bidomain equations) coupled with an ionic model that describes the transmembrane current of the cardiomyocytes. The ionic model is a phenomenological model consisting of a set of coupled non-linear ODEs. The feature selection algorithm proposes a way to compute a so-called composite biomarker for each conductance of interest, designed to perform better in a classification context than classical biomarkers. The composite biomarkers are linear combinations of the entries of a dictionary of features which is given. The calculation of the weights involves Monte-Carlo approximations which use experimental or synthetic (or both) conductances and FP samples. It has been applied to drug classification problems using experimental MEA recordings. The classification was carried out using the Scikit-Learn Python library (Pedregosa et al., 2011) which includes several classification tools. In the present work a Support Vector Classification was used. The data used for the classification consist in FP features extracted from experimental measurements and their associated labels corresponding to the type of drug that is considered.

The purpose of the present work is twofold. First, it intends to establish that the classically used biomarkers may be improved, at least in a classification context, by using composite biomarkers instead. Second, it intends to show that the classification performance may benefit from the addition of synthetic measurements in the calculation of the composite biomarkers. More generally, the authors intend to show that numerical simulations are useful to cardiac electrophysiology in general, beyond the sole scope of drug classification.

First, a comparison between classical and composite biomarkers was carried out. The comparison consists in classifying drugs from experimental measurements using two different strategies: 3v3 and OvA. For each strategy, the classification is performed using classical or composite biomarkers as inputs. As expected, the classification results in both cases are improved when using the composite biomarkers. The latter were indeed designed to be maximally correlated to their associated conductance and minimally correlated to the others. As a consequence, they are more revealing of the underlying conductances than the classical biomarkers. In the 3v3 case, all classification scores significantly increase when using composite biomarkers instead of classical ones. In the OvA case, the improvement is less clear, mainly because most variations in the classification scores are not statistically significant. Nevertheless, the improvement is significant for the *g*_*so*_ AUC and overall the OvA strategy yields better classification results than the 3v3 strategy.

Second, the use of combined experimental and synthetic measurements to compute composite biomarkers is investigated. The composite biomarkers are computed using Monte-Carlo approximations that require conductances and FP features samples. In the previous case, these samples are experimental. The idea is to improve the robustness of the composite biomarkers by incorporating synthetic measurements which better span the parameters (i.e., conductances) space. This approach is meant to compensate the scarcity of experimental data and more generally the fact that the experiments do not cover every possible drug block scenario. The *in silico* measurements allow for a more thorough exploration of the parameter space. Conductances samples were drawn and the computational model was evaluated to generate noisy FPs. From these FPs, the entries of the dictionary of features were computed. The composite biomarkers weights are then computed using a mixed set of experimental and synthetic samples. These composite biomarkers are compared to the ones computed using only experimental data. The same two classification strategies as before are used to compare both approaches. In the 3v3 case, the standard deviation of the Cohen's kappa is significantly decreased, which suggests that this approach makes the classification more robust, at least when considering this metric. The variations of the other classification scores are not statistically significant. In the OvA case, the Cohen's kappa seems to increase in average while its standard deviation decreases. This finding must however be mitigated by the fact that it is not statistically significant. As for the AUC scores, the same observation can be made concerning the *g*_*fi*_ AUC. The standard deviation of the *g*_*si*_ AUC is significantly decreased but the standard deviation of the *g*_*so*_ AUC is increased. Overall, the use of mixed experimental and synthetic measurements seems to improve the classification and make it more robust even though the statistical significance of the results is not conclusive. The use of a larger experimental data set could help assessing the statistical significance of the previous findings.

The use of FP features in a classification context is now discussed. In classification problems, and in machine learning in general, a large number of inputs tend to provoke an over-fitting of the model. This means that the classifier tends to have satisfactory training scores but generalizes poorly on a validation test. This is in part solved by the regularization used but the number of inputs still remains important. When dealing with experimentally recorded FPs, the different signals are often not perfectly synchronized, making timestep-wise comparisons meaningless. Furthermore, an important variability of the signal amplitudes is observed in practice, making even perfectly synchronized signals difficult to compare. Using features extracted from the FP that are do not depend on time shifts and amplitude variations are therefore more robust in a classification context.

### Limitations

The limitations of the proposed approach are now discussed. First we discuss the heterogeneity modeling. In the present work, we make the assumption that the hiPSC-CM medium is a continuous mixture of two cell types (“A” and “B”) based on a ventricular endocardium cell model, modified to match the action potential duration of the experimental recordings. The actual nature of the hiPSC-CM types is still quite unknown, to the authors knowledge, even though some studies suggest it is a mixture of atrial, ventricular and pacemaker cells (Matsa et al., 2011). Even if the medium can be well characterized in a particular setting, it varies greatly from one cell line to another. In the present work, we propose a general method to generate heterogeneous media and for the sake of simplicity we restricted our study to a continuous mixture of two cell types. The approach is easily generalizable to more realistic heterogeneities, including for instance atrial, ventricular and pacemaker cells. Second, the conductances values associated with the experimental measurements are not known and are therefore approximated using Equation (2). This approximation is, however, subject to several sources of uncertainty such as the IC_50_ whose value for a given drug may vary according to the source considered (Mirams et al., 2011; Kramer et al., 2013). The uncertainties also come from the Hill's equation which is an imperfect model. Knowing the exact values for the conductances is, however, not critical since those values are only needed to derive the composite biomarkers and are not directly used during the classification procedure. Furthermore, the drugs studied in the present work are assumed to be single channel blockers. In reality, some drugs (e.g., diltiazem) are known to target more than one ion channel. In fact, it can be considered that any drug affects every ion channel with different IC_50_ values. In the present work, we make the strong assumption of single channel blocking as a first step toward a finer description of the drugs effects. This assumption is also motivated by the simplicity of the considered ionic model which only counts three different currents. Note also that mexiletine primarily blocks the late sodium channel current and not the fast one. In the MV model, there is no distinction between these two currents.

Another limitation comes from the computational model used in the present work. The sources of error are multiple: space and time discretizations, conductivities errors, modeling errors, etc. These errors are not critical either since the computational model is only used to compute the composite biomarkers weights. This study shows that, despite the modeling errors, adding synthetic measurements simulated by the computational model leads to a better and more robust classification. In the present study, we based our *in silico* modeling on the MV ionic model. It is a very simplistic model which is not able to reproduce complex behaviors such as early after depolarizations for instance. Furthermore, the hiPSC-CM are spontaneously excitable cells in our case while the MV model is not sophisticated enough to reproduce such a behavior. For this reason, it is not suited to the study of drug arrhythmogenicity. However, in the scope of the present work, we have established that it is suited to the characterization of drug-induced channel block, at least for a coarse description of it. Furthermore, it was also established in Raphel et al. (2017) that it is possible to identify which of the three main currents is affected by a drug using the MV model. Other limitations come from the classification strategies. Both classification strategies are non-exhaustive in that they do not explore every possible way of splitting the data set. Furthermore, the classification metrics used to compare the different approaches are not flawless. In some cases comparing AUCs for instance is not the best way to compare classifiers (Adams and Hand, 2000). Other metrics exist, such as the mean squared error, but were not investigated in this work. Finally, the composite biomarkers derived in the present work are not optimal in the sense that their correlation with their associated conductances is not equal to one, as seen in Figure 8.

The limitations of the study also arise from the MEA measurements themselves. Variations of the repolarization wave morphology and the depolarization amplitude from one experiment to another constitute a technical challenge when one tries to extract meaningful information from the measurements. In the present study, we propose to model the heterogeneities of the experimental settings (CM cell types and stimulation location) to account for the observed variability in the data. Furthermore, considering ratios of biomarkers with their control counterparts makes the approach more robust and less dependent on fluctuations from one experiment to another.

### Perspectives

We now discuss some perspectives that could lead to interesting future works. Other classification methods than SVC exist, such as neural networks or random forests for instance. It would be interesting to assess whether the findings of this work are still valid when considering other classification tools. It would also be interesting to evaluate which classification tool generally performs best in the present drug classification context. Other perspectives concern the composite biomarkers computed using a mixed set of synthetic and experimental measurements. In the present work, the mixed set is roughly composed of half synthetic and half experimental measurements. However, other proportions could be investigated and an optimal proportion with respect to the classification score could be found. In the present work, only sodium, potassium and calcium antagonists drugs are considered but other types of drugs exist. Drugs that affect other ionic channels or even simultaneously several of them could be investigated. In parallel, more sophisticated ionic models including more current types would need to be used to model these new drugs. This would, of course, come at the expense or more computationally intensive simulations. Another interesting perspective would be to train the classifiers with only synthetic measurements instead of experimental ones. This would be very useful when experimental data are insufficient or even not available. The classifiers could also be trained with a mixed set of synthetic and experimental data just like it is done in this work for the computation of composite biomarkers. Finally, as explained earlier, the point of the present work is not the direct assessment of drugs arrhythmogeneicity but rather the identification of the main channel block induced by the drugs. This is, in the author's opinions, a necessary first step toward a better understanding of MEA measurements and *in fine* its use in drug safety evaluation. Considering a larger set of drugs and more realistic ionic models in order to perform drugs classification based on their arrhythmogenicity (or TdP risk) will be the purpose of future works.

## Ethics statement

The MEA recordings were made using a commercially available line of hiPSC-CM provided by the CDI (Cellular Dynamics International) company.

## Data accessibility

The implementation (in Python) of the composite biomarkers algorithm is available at this URL: https://github.com/eltix/numbio.

## Author contributions

All authors listed have made substantial, direct and intellectual contribution to the work, and approved it for publication.

### Conflict of interest statement

The authors declare that this study received funding from Instem. The funder was not involved in the study design or collection, analysis, or interpretation of the data.

## Footnotes

^1^Systems, M. Microelectrode array (mea) manual. http://www.multichannelsystems.com/sites/multichannelsystems.com/files/documents/manuals/MEA_Manual.pdf.
