# Supplementary material for: Composite Biomarkers Derived from Micro-Electrode Array Measurements and Computer Simulations Improve the Classification of Drug-Induced Channel Block
Source: Front Physiol. 2018 Jan 4;8:1096. doi: 10.3389/fphys.2017.01096 (PMC5762138; doi:10.3389/fphys.2017.01096)
Supplement: Supplementary file 1 [file Presentation1.PDF]

Supplementary Material

Composite biomarkers improve classification of drug-induced channel block

1 Minimal Ventricular model parameters

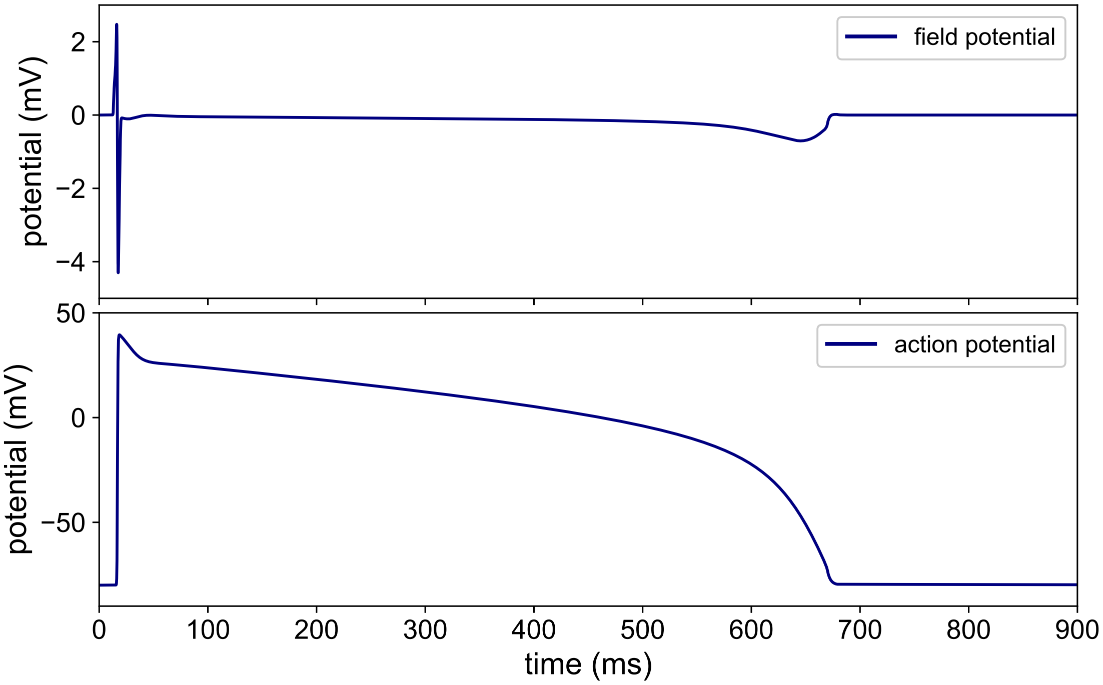

Figure 1: Simulated field potential and action potential recorded on the same electrode with the same time scale.

Parameter name	cell type A ($c = 0$)	cell type B ($c = 1$)
τ_{v1}^{-}	150	391
τ_{v2}^{-}	20	34.8
τ_{v}^{+}	2.90	10.6
τ_{w1}^{-}	12	1.12
τ_{w2}^{-}	280	35.9
τ_{w}^{+}	560	510
τ_{fi}	0.111	0.343
τ_{o1}	235	441
τ_{o2}	3	4.50
τ_{so1}	20	176
τ_{so2}	0.6	0.163
τ_{s1}	5.47	0.4
τ_{s2}	4	21.2
τ_{si}	1.45	8.83
$\tau_{w\infty}$	0.027	0.018
θ_{v}	0.3	0.00489
θ_{w}	0.13	0.0459
θ_{v}^{-}	0.2	0.149
θ_{o}	0.006	0.00476

Table 1: Minimal Ventricular model parameters calibrated to qualitatively replicate field potential experimental measurements.

2 Imperfect electrode model

The bidomain model describes the evolution of the transmembrane potential V_m and the extracellular potential ϕ_e in a domain Ω. We denote by R_i, R_{el} and C_{el}, the internal resistance of the measurement device, the electrode resistance and the electrode capacitance respectively. The field potential ϕ_f^k measured on an electrode e_k is given by $\phi_f^k = R_i I_{el}^k$, where I_{el}^k is linked to the averaged extracellular potential $\phi_{e,mean}^k$ at the electrode e_k by the equation:

$$\frac{dI_{el}^k}{dt} + \frac{I_{el}^k}{\tau} = \frac{C_{el}}{\tau}\frac{d\phi_{e,mean}^k}{dt}, \tag{1}$$

where $\tau = (R_i + R_{el})C_{el}$.

For the present study the parameters values are summarized in Table 2.

C_{el}	R_i	R_{el}
$1nF$	$2M\Omega$	$10M\Omega$

Table 2: Parameters used for the imperfect electrode model.

The equivalent electrical circuit is represented in Figure 2.

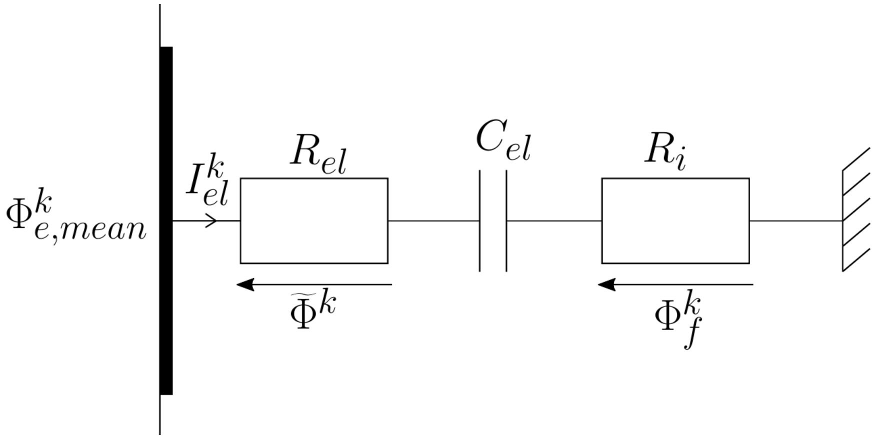

Figure 2: Electrode model electrical circuit.

3 Classification metrics

We now present the different classification metrics used in this work.

Cohen's kappa Cohen's kappa, denoted by κ, is particularly suited for multi-class and/or imbalanced classification problems. The main idea is that it measures the labeling discrepancy between two annotators (or classifiers). It is simply adapted to our case by considering one of the annotators as the ground truth (true labels). Its formula reads:

$$\kappa = \frac{p_o - p_e}{1 - p_e}, \tag{2}$$

where p_o is the observed agreement between the two annotators and p_e is the probability of an agreement between two random annotators. For further details, the reader is referred to Scikit-learn's implementation [1] of Cohen's kappa.

ROC The receiver operating characteristic area under curve (ROC AUC, later referred to as AUC for the sake of clarity) is basically associated with binary classification problems. In our case, one can define an AUC for each class k by considering all the other classes as only one class. With SVC it is possible to evaluate, in addition to the predicted class, the probability of belonging to each class. Given a threshold parameter (that varies between 0 and 1), it is possible to decide if a sample belongs to a given class when the SVC probability returned for this class is greater than the threshold parameter. The predicted class therefore depends on this parameter. When all samples of the validation set have been tested, the following quantities are computed, for each class k and for a given threshold parameter:

- true positives (TP): number of samples affected to class k which are indeed in class k.
- false positives (FP): number of samples affected to class k which are actually *not* in class k.
- true negatives (TN): number of samples affected to another class than k which are indeed *not* in class k.
- false negatives (FN): number of samples affected to another class than k which are actually in class k.
- true positive rate (TPR): $\frac{TP}{TP+FN}$.
- false positive rate (FPR): $\frac{FP}{FP+TN}$.

The ROC is the curve of TPR against FPR as the threshold parameter varies between 0 and 1. The AUC is simply the area under this curve. The AUC ranges from 0 to 1. An AUC of 0.5 is equivalent to a coin flip, meaning the classifier has no predictive power. An AUC of 1 corresponds to the perfect classifier: each sample has been correctly classified. An AUC below 0.5 corresponds to a bad classifier: it does worse than a coin flip.

4 Additional classification results

[1] https://github.com/scikit-learn/scikit-learn/blob/ab93d65/sklearn/metrics/classification.py#L278

Score		Splitting index (fold)							
		1	2	3	4	5	6	7	8
3v3	Cohen's kappa	0.46	0.05	0.46	0.04	0.37	0.24	0.35	0.22
	g_{fi} AUC	0.69	0.72	0.69	0.73	0.59	0.99	0.55	0.99
	g_{si} AUC	0.98	0.97	0.98	0.97	0.99	0.98	0.98	0.98
	g_{so} AUC	0.74	0.68	0.75	0.68	0.64	0.72	0.66	0.70
OvA	Cohen's kappa	0.44	0.06	0.45	0.06	0.73	0.54	0.73	0.49
	g_{fi} AUC	0.79	0.83	0.79	0.81	0.73	0.99	0.74	0.99
	g_{si} AUC	0.86	0.97	0.85	0.97	1.00	0.73	1.00	0.74
	g_{so} AUC	0.76	0.70	0.74	0.69	0.69	0.99	0.61	0.98

Table 3: Classification scores using classical biomarkers.

Score		Splitting index (fold)							
		1	2	3	4	5	6	7	8
3v3	Cohen's kappa	0.00	0.42	0.57	0.60	0.87	0.57	0.86	0.56
	g_{fi} AUC	0.75	0.97	0.76	0.92	0.89	0.99	0.93	0.99
	g_{si} AUC	1.00	1.00	1.00	1.00	1.00	1.00	1.00	1.00
	g_{so} AUC	0.79	0.84	0.79	0.82	0.85	0.93	0.88	0.87
OvA	Cohen's kappa	0.88	0.29	0.69	0.29	0.71	0.57	0.72	0.15
	g_{fi} AUC	0.87	0.35	0.70	0.36	0.94	0.99	0.75	0.99
	g_{si} AUC	0.83	0.84	0.86	0.97	1.00	1.00	1.00	0.99
	g_{so} AUC	1.00	0.84	1.00	0.78	0.99	0.87	0.99	0.88

Table 4: Classification scores using composite biomarkers computed from experiments only.

	classical biomarkers		composite biomarkers	
Score	mean	std.	mean	std.
Cohen's kappa	0.25	0.17	0.54	0.27
g_{fi} AUC	0.78	0.14	0.91	0.10
g_{si} AUC	0.99	0.00	1.00	0.00
g_{so} AUC	0.71	0.05	0.85	0.07
averaged AUC	0.83	-	0.92	-

Table 5: Comparison between classical and composite biomarkers with the 3v3 classification strategy with a half data set.

	classical biomarkers		composite biomarkers	
Score	mean	std.	mean	std.
Cohen's kappa	0.37	0.25	0.54	0.23
g_{fi} AUC	0.82	0.10	0.73	0.26
g_{si} AUC	0.90	0.10	0.95	0.07
g_{so} AUC	0.79	0.12	0.92	0.08
averaged AUC	0.83	-	0.87	-

Table 6: Comparison between classical and composite biomarkers. Classification scores in the one-vs-all scenario with a half data set.

Score		Splitting index (fold)							
		1	2	3	4	5	6	7	8
3v3	Cohen's kappa	0.57	0.62	0.57	0.51	0.49	0.57	0.83	0.57
	g_{fi} AUC	0.84	0.85	0.79	0.85	0.94	0.92	0.95	0.95
	g_{si} AUC	1.00	1.00	1.00	1.00	1.00	1.00	1.00	1.00
	g_{so} AUC	0.90	0.79	0.80	0.79	0.94	0.81	0.94	0.87
OvA	Cohen's kappa	0.77	0.40	0.67	0.41	0.85	0.57	0.92	0.48
	g_{fi} AUC	0.66	0.67	0.71	0.67	1.00	0.93	0.89	0.97
	g_{si} AUC	0.97	0.98	0.99	0.97	1.00	0.99	1.00	1.00
	g_{so} AUC	0.96	0.85	0.50	0.85	1.00	0.63	1.00	0.69

Table 7: Classification scores using composite biomarkers computed from combined experiments and simulations.
