# Supplementary material for: Composite Biomarkers Derived from Micro-Electrode Array Measurements and Computer Simulations Improve the Classification of Drug-Induced Channel Block
Source: Front Physiol. 2018 Jan 4;8:1096. doi: 10.3389/fphys.2017.01096 (PMC5762138; doi:10.3389/fphys.2017.01096)
Supplement: Supplementary file 2 [file DataSheet1.pdf]

APPENDIX: COMPUTATION OF THE BIOMARKERS

In this section, we provide details on how to compute the biomarkers from FP time series as shown in Figure 2. For a given signal, we denote by t the time vector and y the FP. Each signal is divided into two parts: the depolarization region (t_1, y_1) from $t = 0$ to $t = 100$ ms and the repolarization region (t_2, y_2) from $t = 100$ to $t = 1200$ ms.

Depolarization amplitude (DA)

The DA is simply defined as the difference between the maximum and minimum values of the potential during the depolarization:

$$\text{DA} = \max(y_1) - \min(y_1). \tag{17}$$

Depolarization width (DW)

The DW is defined as the total duration during which the field potential is above, in absolute value, 10% of its peak value:

$$\text{DW} = \int_{t_1 \in I} \mathrm{d}t_1, \tag{18}$$

where $I = \{t_1 \mid \quad |y_1(t_1)| \geq 0.1 \times \max(|y_1|)\}$

Repolarization amplitude (RA)

The RA is defined as the maximum (in absolute value) of the repolarization.

$$\text{RA} = \max(|y_2|). \tag{19}$$

Field potential duration (FPD)

The FPD is defined as the time difference between the maximum (in absolute value) of the depolarization and the maximum (in absolute value) of the repolarization. Let $t_d = t\left[\underset{t}{\operatorname{argmax}}(|y_2(t)|)\right]$ and $t_r = t\left[\underset{t}{\operatorname{argmax}}(|y_1(t)|)\right]$. Then,

$$\text{FPD} = t_r - t_d. \tag{20}$$

Area under the curve of the repolarization wave (AUCr)

The AUCr is defined as the area under the curve of y_2 truncated around $\pm\Delta t$ of t_r. We used $\Delta t = 100$ms. The integral is approximated using the trapezoidal rule.

$$\text{AUCr} = \left| \int_{t_r - \Delta t}^{t_r + \Delta t} y_2(t) \mathrm{d}t \right| \tag{21}$$

Repolarization center (RC)

The RC is defined as the offset of the barycenter (with respect to time) of the repolarization wave.

$$\text{RC} = \int_{t_r-\Delta t}^{t_r+\Delta t} t\bar{y}_2(t)\mathrm{d}t - t_d, \tag{22}$$

where $\bar{y}_2(t)$ is a non-dimensional rescaling of $y_2(t)$ so that it is strictly positive and integrates to 1 on $[t_r - \Delta t, t_r + \Delta t]$. $\bar{y}_2(t)$ may be seen as a probability density function over the domain $[t_r - \Delta t, t_r + \Delta t]$.

Repolarization width (RW)

The RW is defined as the standard deviation of the repolarization wave.

$$\text{RW} = \left[\int_{t_r-\Delta t}^{t_r+\Delta t} t^2\bar{y}_2(t)\mathrm{d}t - \left(\int_{t_r-\Delta t}^{t_r+\Delta t} t\bar{y}_2(t)\mathrm{d}t\right)^2\right]^{1/2}. \tag{23}$$

Field potential notch (FPN)

The FPN is defined as the potential value 4ms after t_d. The FPN value is smoothed out by multiplying the signal with a test function and then integrates the product. This proves to be less sensitive to noise than just a point-wise evaluation. Let $\phi(t_1) = \exp\left[-\frac{(t_1-(t_d+4))^2}{.04}\right]$. Then,

$$\text{FPN} = \int_{t_1} y_1(t_1)\phi(t_1)\mathrm{d}t_1. \tag{24}$$

Conduction velocity (CV)

The CV is defined as the velocity of the depolarization wave. Since there are 9 electrodes in the studied MEA device, one can identify the first electrode, denoted by e_a, to detect the depolarization peak at time t_a and the last one, denoted by e_b, at time t_b. The velocity is then defined as follows:

$$\text{CV} = \frac{\sqrt{(x_{e_b} - x_{e_a})^2 + (y_{e_b} - y_{e_a})^2}}{t_b - t_a}. \tag{25}$$

Note that this is the only biomarker for which we do not take statistics (median, mean, etc.) over the electrodes since there is only one value for all nine electrodes.
